# A Cluster-Randomized Trial to Test Sharing Histories as a Training Method for Community Health Workers in Peru

**DOI:** 10.9745/GHSP-D-19-00332

**Published:** 2020-12-23

**Authors:** Laura C. Altobelli, José Cabrejos-Pita, Mary Penny, Stan Becker

**Affiliations:** a Future Generations University, Franklin, WV, USA.; b Future Generations, Lima, Peru.; c Universidad Peruana Cayetano Heredia, Lima, Peru.; d Superintendencia Nacional de Salud, Lima, Peru.; e Nutrition Research Institute, Lima, Peru.; f Johns Hopkins Bloomberg School of Public Health, Baltimore, MD, USA.

## Abstract

**Background:**

Community health workers (CHWs) are increasingly deployed to support mothers’ adoption of healthy home practices in low- and middle-income countries. However, little is known regarding how best to train them for the capabilities and cultural competencies needed to support maternal health behavior change. We tested a CHW training method, Sharing Histories (SH), in which CHWs recount their own childbearing and childrearing experiences on which to build new learning.

**Methods:**

We conducted an embedded cluster-randomized trial in rural Peru in 18 matched clusters. Each cluster was a primary health facility catchment area. Government health staff trained female CHWs using SH (experimental clusters) or standard training methods (control clusters). All other training and system-strengthening interventions were equal between study arms. All CHWs conducted home visits with pregnant women and children aged 0–23 months to teach, monitor health practices and danger signs, and refer. The primary outcome was height-for-age (HAZ)<−2 Z-scores (stunting) in children aged 0–23 months. Household surveys were conducted at baseline (606 cases) and 4-year follow-up (606 cases).

**Results:**

Maternal and child characteristics were similar in both study arms at baseline and follow-up. Difference-in-differences analysis showed mean HAZ changes were not significantly different in experimental versus control clusters from baseline to endline (*P*=.469). However, in the subgroup of literate mothers, mean HAZ improved by 1.03 on the Z-score scale in experimental clusters compared to control clusters from baseline to endline (*P*=.059). Using generalized estimating equations, we demonstrated that stunting in children of mothers who were literate was significantly reduced (Beta=0.77; 95% confidence interval=0.23, 1.31; *P*<.01), adjusting for covariates.

**Conclusion:**

Compared with standard training methods, SH may have improved the effectiveness of CHWs as change agents among literate mothers to reduce child stunting. Stunting experienced by the children of illiterate mothers may have involved unaddressed determinants of stunting.

## Abstract (precis)

Women naturally communicate using life narratives. Through systematic recall and sharing memories of their own childbearing and child rearing experiences, community health workers (CHWs) become engaged and empowered to change their own and other mothers’ health behaviors. Training CHW with sharing histories can improve capabilities as change agents for better child health.

## Teaser

**Key Findings:**

- Sharing Histories method for training community health workers (CHWs) was associated with reduced child stunting compared with a standard CHW training method.
- The training method’s impact on child stunting was not present when mothers were illiterate.

**Key Implications:**

- CHWs can learn better by using Sharing Histories as the basis for their training; then, they can use the same method to better help mothers change behaviors.
- Program managers can use this training methodology to strengthen capacities of CHW trainers to improve the cultural literacy of trainees.
- Primary health care personnel who train CHWs can identify local cultural and social norms when CHWs share their experiences of childbearing and childrearing.
- Primary health care personnel can easily apply this CHW training method to develop social bonds with CHWs, address CHW cultural competencies, and provide CHWs with a strategy to effectively discuss sensitive culturally determined behaviors with mothers.

Resumen en español al final del artículo.

## INTRODUCTION

Supporting mothers to adopt healthy home practices could be one of the keys to improving child health. Health behavior change strategies are frequently used in global health, but the evidence of their effect on health outcomes remains unclear due to numerous pitfalls in their design and evaluation.^1^ A central challenge for these strategies is how to effectively help mothers gain knowledge and change behaviors in communities with strong traditional beliefs and poor access to health information. A worldwide priority for child health and development is the reduction of chronic child malnutrition (poor linear growth or stunting), which arises from a broad range of causes related to home practices for maternal nutrition, breastfeeding and weaning, water, sanitation, hygiene, and infection prevention. Peru is a low- to middle-income country (LMIC) that has seen a major overall reduction in stunting since 2008, but high rates persist in mountain and jungle regions where numerous cultural practices negatively influence maternal and child health.^2^ ^,^ ^3^

Community health workers (CHWs) are a global priority to help reach impact and equity goals through universal health coverage and Sustainable Development Goals.^4^ ^–^ ^8^ CHWs are the lowest level of frontline health workers and are frequently volunteers, delivering a wide range of services in homes and communities including health education and support on nutrition, malaria, tuberculosis, HIV/AIDS, sexually transmitted infections and noncommunicable disease, preventive maternal and reproductive health services in the home, management of uncomplicated childhood illnesses, and access to services, among others.^7^ ^,^ ^9^ ^,^ ^10^ CHWs can be critical actors for reporting maternal and perinatal deaths occurring in the community.^11^ We know some of what works, but a large gap remains between that knowledge and “how to make it work.” An estimated 5 million CHWs are deployed worldwide,^5^ but their effectiveness and linkage to health subsystems within their communities vary.^12^ The World Health Organization (WHO) identifies CHWs as important to their Global Strategy for Human Resources,^13^ but implementation research on CHW programs is needed.^14^ ^,^ ^15^

To be effective, CHW programs should have detailed plans for governance/management, selection, training, supervision, engagement with communities, relationship with the health system, scaling up, and monitoring and evaluation.^16^ ^–^ ^20^ In this study, we highlight the need for the identification and testing of the best methods to train CHWs.^7^ ^,^ ^21^ ^–^ ^23^ We have not found other reports with results on comparative studies. We submit that special training methods are needed to adequately prepare CHWs to support home behavior change. Behavior change theories developed for industrialized countries often cannot be applied in areas of LMICs with embedded cultural beliefs, attitudes, and practices.^24^ More recently, integrated behavior change models for LMICs have been developed using a mix of theories and strategies.^25^ ^,^ ^26^ Even though qualitative research can identify the specific beliefs that impede the home practice of key health behaviors, defining the “black box” of how and why mothers, families, and communities hold onto cultural beliefs is a major challenge when working to empower change agents who can convince mothers to change cultural practices.^27^

We highlight the need for the identification and testing of the best methods to train CHWs.

An important part of empowering CHWs is helping them build their own self-confidence and agency so they can thus empower mothers. These are key dimensions of maternal capabilities needed to implement new knowledge of proper child care.^28^ ^,^ ^29^ We assume that CHW efforts empower communities,^9^ but it is less commonly recognized that CHWs themselves need to become empowered to be the change agents needed to support mothers and families for active self-care. According to Kane and colleagues, “… to be able to empower the communities they serve, we argue, it is essential that CHWs themselves be, and feel, empowered….”^30^ A review of randomized controlled trials with a “realist” approach concluded that interventions by CHWs worked if there was a “… sense of relatedness with beneficiaries and public services; increase in self-esteem; sense of self-efficacy….”^31^ The same author concluded that if these factors were absent, CHW performance would be negatively affected even with the same interventions.^31^ Some researchers have suggested the existence of a “secret sauce” that would help to empower women with knowledge, motivation, and increased self-efficacy even when scaling up community strategies into government programs. This “sauce” could be the next breakthrough to sustainably improve maternal, newborn, and child health behaviors.^32^

CHWs themselves need to become empowered to be the change agents needed to support mothers and families for active self-care.

How to maintain fidelity of empowerment and behavior change approaches in the scale-up of interventions with CHWs remains a key challenge.^33^ As a part of being and feeling empowered, the cultural competency of health workers and CHWs is an essential skill to reach patients of diverse cultures to improve their health literacy.^34^ Scaling up CHW programs in government systems likely relies in part on how well health providers in primary health care (PHC) services can serve as trainers to facilitate CHW learning. They are generally not educators, and they often rely on medical terminology and heuristic methods to train CHWs. Methods for teaching CHWs should be adapted to their educational level, which is very often the same as that of the mothers with whom they will work. The train-the-trainer model, CHW training materials, incentives, and supportive supervision are factors that have yet to be refined in government systems as well as nongovernmental organization and other private sector efforts to support CHWs.

This study provides a new focus in the development of empowerment and cultural competency by testing a training method that builds on cultural beliefs and practices of CHWs so they increase their self-esteem and have a greater sense of interpersonal relatedness with households and the health system. From this foundation, they can become more effective behavior change agents within traditional populations.

We conducted a cluster-randomized controlled trial of our new teaching strategy called Sharing Histories (SH) in rural Peru to test the impact on child stunting when female CHWs are trained with this method. We hypothesized that mothers would be more likely to change health knowledge and behaviors, and their children would consequently have better growth, if the mothers received health information from CHWs trained with the SH method, compared with the situation in which health education interventions were received from CHWs trained with standard methods.

The method is used to train CHWs and community supervisors (CSs), as well as to provide direct education and counseling of mothers, and it can be implemented at low cost through the government PHC system. The training method is oriented toward enhancing CHWs’ empowerment, as well as their knowledge and skills, as community change agents.

The Sharing Histories training method is oriented toward enhancing CHWs’ empowerment, as well as their knowledge and skills, as community change agents.

## SHARING HISTORIES TRAINING METHOD

The SH teaching method is a key component of an integrated teaching strategy we developed to improve maternal, neonatal, and child health. It was piloted in Afghanistan, India, and Peru.^35^ ^–^ ^38^

Research on autobiographical memory explains how the Sharing History method helps CHWs learn to effectively convince other women to change behaviors.^38^ ^,^ ^39^ The utility of memory fits into 3 categories: self function (continuity of the self), social function (developing and maintaining social bonds), and directive function (guiding present and future behaviors).^40^ ^,^ ^41^ The latter 2 functions are relevant to SH. The social function creates bonding and trust among CHWs who share their childbirth and childrearing memories with each other, and these memories are then used by trainers as the basis for learning. The directive function has been found to influence future behavior.^40^ Neuroscience research suggests that physical consolidation of neurons occurs when memory is stimulated and new information is added.^42^

A manual provides details of the SH training method and the community health model (Box).^43^

BOX Teaching Strategy of Sharing Histories

The teaching strategy of SH builds on CHWs sharing their personal experiences and actions regarding their pregnancies, births, postpartum periods, care and feeding of newborns and infants, and events surrounding any sickness or death. Monthly full-day workshops are held in the primary health care facility. Each of the 7 training module topics has a series of class sessions with 6 steps:

1. If pregnancy is the day’s topic, each CHW shares her pregnancy experiences while the trainers or assistants take notes on the history format.
2. Trainers list key actions mentioned by CHWs, then lead CHWs through a guided discussion of each action, using colors to indicate whether the action is beneficial, neutral, or potentially harmful and then discussing why.
3. Then, picture cards on key practices are used to teach each best practice in a class session, referring to the CHWs’s shared experiences and further asking about and analyzing local customs related to each best practice.
4. Participatory methods are then used to practice what is learned in each class session, such as sociodramas of home visits to teach mothers using the same methods and materials, monitor practices and danger signs, and make referrals.
5. Each class session ends with participatory evaluation of learning, using games and exercises.

Between monthly class sessions, each CS meets with 5 or 6 of her assigned CHWs to review the monthly topic, and CHWs practice using the respective flip charts and checklists for monitoring key maternal health practices and danger signs.

CHW trainees can take ownership of and learn from their own cultural beliefs and practices, which empowers them to change their practices and helps them become more capable and convincing promoters of the same key behavioral changes with mothers in their communities. The process of sharing personal experiences could have several benefits including providing CHWs, who may be timid about speaking in a group, with opportunities to practice verbal expression and revealing cultural practices that can be used to build new learning that would otherwise only be identified through expensive and time-consuming qualitative research.

## METHODS

### Project Intervention

**FIGURE 1.**
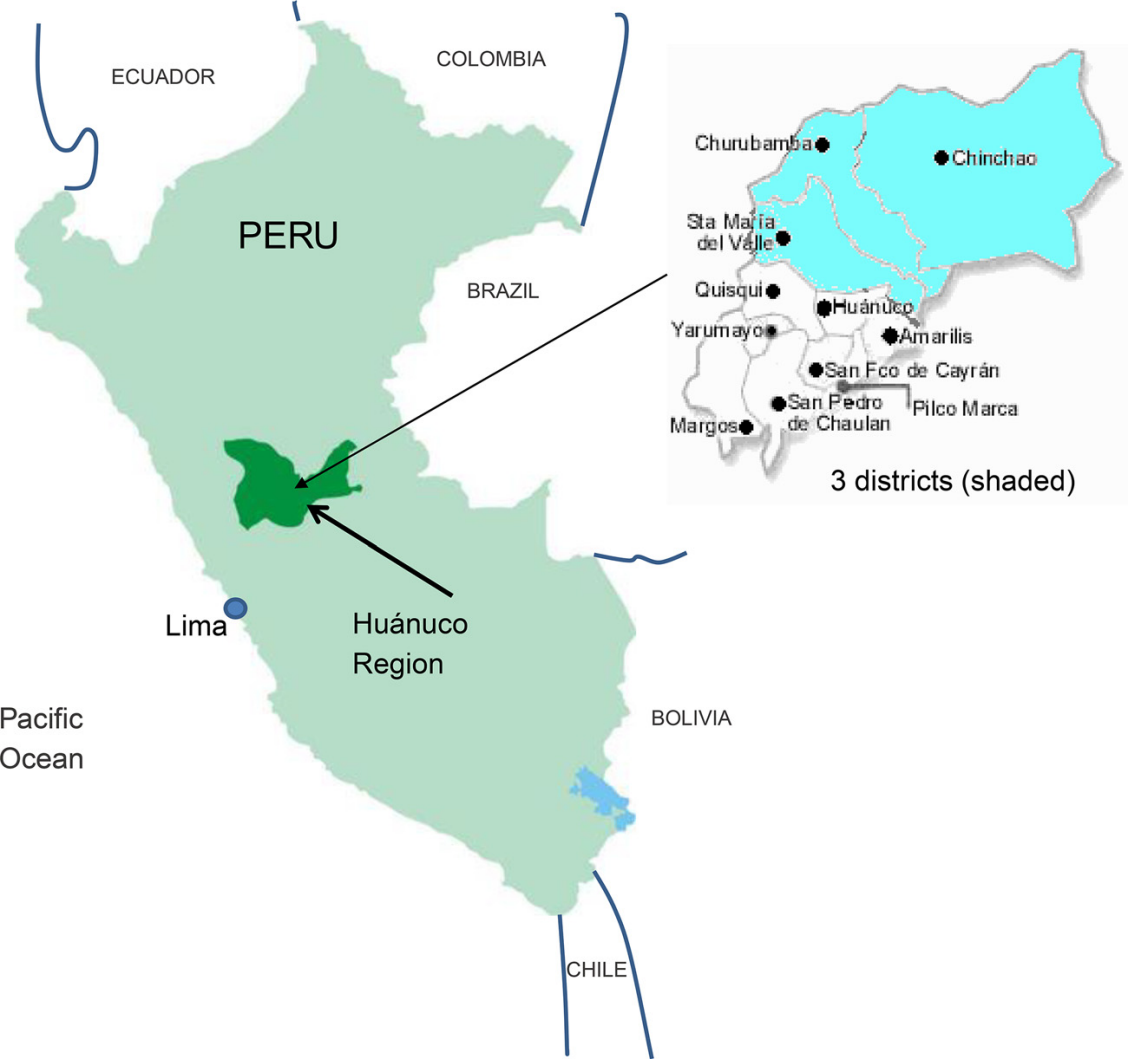
Location of Rural Districts, Huánuco Region, Peru, Where Cluster-Randomized Controlled Trial of Community Health Worker Training Methodology Was Conducted

This study was an embedded component of a larger integrated project intervention called Health in the Hands of Women, which was aimed at reducing chronic child malnutrition by linking strengthened PHC services and district government to a sustainable community-oriented system to support maternal behavior change for improved maternal, neonatal, and child health.^44^ The area where this study was conducted included 3 rural districts with 82,000 inhabitants in the area of the upper Huallaga River on the eastern slope of the Andes mountains in the Huánuco Region of Peru (Figure 1). In both experimental and control study arms, we implemented interventions to strengthen capabilities and processes so that district government, health services, and local health administration committees^45^ could better support CHWs. The methodology of CHW training was the only factor that varied between the 2 study arms (Table 1).

**TABLE 1.** Comparison of CHW Training Methodology Interventions Used in a Cluster-Randomized Controlled Trial, Huánuco Region, Peru

**Interventions**	**Experimental Clusters**	**Control Clusters**
Basic strengthening of primary health care services: orientation to community health strategies, interdisciplinary team building for health staff, self-assessment, and planning for community health actions	Yes	Yes
Basic strengthening of local government to support community MNCH, to gain their commitment to provide financial and incentive support to CHWs and CSs	Yes	Yes
Training of facilitators (health personnel trainers) on adult education methods	Yes	Yes
Provision of a complete set of 7 flip charts to each CHW and CS	Yes	Yes
Training of facilitators on use of facilitator manuals based on Sharing Histories as the CHW training method	Yes	No
Training of facilitators on use of facilitator manuals based on a standard CHW training method	No	Yes
Continuous monthly training of CHWs and CSs using Sharing Histories as the training method	Yes	No
Continuous monthly training of CHWs and CSs using a standard CHW training method	No	Yes
Monthly home visits conducted by CHWs, supported by CSs and health staff to educate mothers, monitor MNCH behaviors, identify danger signs, and refer to the health facility	Yes	Yes
Monthly supervision of CHWs by CSs	Yes	Yes

Abbreviations: CHW, community health workers; CS, community supervisors; MNCH, maternal, neonatal, and child health.

As part of the larger project, selection criteria, roles, and tasks were clearly defined for each type of human resource for community health: CHWs, CSs, and PHC facility staff who train CHWs and CSs (Table 2).

**TABLE 2.** Human Resources With Roles and Tasks for Community Promotion of Maternal, Neonatal, and Child Health, Huánuco Region, Peru

**CHW**	**CS**	**Facilitators (Trainers)**
**Selection process**		
Selected preferably by other women in the community	Selected by a panel of judges from the local PHC facility and municipal government	Self-selected with the approval of their superior
**Selection criteria**		
Respected older woman with grown children	Female, literate, at least 5 years of prior CHW experience or auxiliary nurse training; ability to work half-time and travel between communities	Health professional; preferably woman who has a long-term contract in PHC facility
**Workload**		
1 CHW for every 30 families (on average, 2 or 3 pregnant women and 2 or 3 children aged 0–23 months)	1 CS supports 10–15 female CHWs	1 trainer per group of 10–25 CHWs and their respective CS
**Key roles**		
Attend monthly 1-day trainings at nearest PHC facility Meet monthly in small groups with her CS for reinforcement of training and practice with flip charts and monitoring formats Create a map of her 30 households, identifying pregnant women and children aged 0–23 months Visit each pregnant woman and child aged 0–23 months on a monthly basis Fill out simple monitoring checklists, referral slips, and monthly activity report checklists	Ensure that her assigned CHWs attend month training sessions in the PHC facility Meet with her CHWs in small groups of 5–7 CHWs once or twice a month to review the training from the latest workshop in the PHC facility and to practice using flip charts to teach mothers Accompany CHWs on home visits until the CHW feels comfortable visiting alone Attend monthly training workshops along with CHWs in the PHC facility	Organize and hold 1 monthly workshop for CHWs in their own PHC facility Train CHWs and CSs on how to educate and monitor mothers in the home using the flip chart series and monitoring tools, following a facilitator manual corresponding to each flip chart Receive training in how to utilize the facilitator manual that accompanies each of 7 flip charts Use the Sharing Histories teaching methodology as incorporated into each facilitator manual
**Key tasks during monthly home visits to pregnant women and children aged 0–23 months** Share histories and teach mothers using flip charts by stage of pregnancy or child age Monitor health practices and record on pictorial checklists by stage of pregnancy or child age Observe for danger signs using pictorial checklists by stage of pregnancy or child age Make referrals using pictorial referral slips for maternal-child preventive care and when danger signs are detected		
**Incentives**		
In-kind from the health system: certificate of recognition, training, supervision visits by CSs In-kind from district government: clothing items identifying her as a CHW or CS with name of the district, a food basket and party for annual Health Promoters’ Day and Christmas	Monthly stipend equivalent to about one-third the salary of an auxiliary nurse (To ensure accountability, monthly payment from district government was based on demonstrated completion of the 4 key roles) In-kind incentives from both the health sector and municipality, the same as for female CHWs	Training and recognition

Abbreviations: CHW, community health workers; CS, community supervisors; PHC, primary health care.

Key project messages for training CHWs and CSs and for teaching mothers were identified from best-practice literature on reducing child stunting and from our baseline qualitative studies on local practices of the target population. Key messages were delineated in a series of 7 flip charts focused on 7 areas of maternal knowledge and practice during the first 1,000 days from conception. These 7 flip charts covered the topics: pregnancy, birth and postpartum, newborns, breastfeeding, child growth and nutrition, infant diarrhea, and infant pneumonia. Each flip chart emphasized home practices, preventive care services, and recognition of danger signs for which medical care should be sought. Key messages on water, sanitation, and hygiene (WASH) practices were included throughout the 7 flip charts. The flip chart messages and artwork were previously developed and validated by the research team in another rural area of Peru (Cusco)^46^ with artwork adapted to reflect local clothing and hair styles of Huánuco. The breastfeeding flip chart was adapted from one previously developed in Lima.^47^

All trainers, CSs, and CHWs received the flip charts, which were identical for both study arms.

For the trainers, 2 sets of step-by-step training manuals were developed so they could apply different methods for training CHWs on the flip chart messages: (1) a set that incorporated the SH teaching method and (2) a set that used standard participatory CHW training methods. CHWs in both study groups received and studied the same flip charts and used them in home visits to teach mothers.

A set of 12 checklists and reporting formats were other key tools taught to and used by CHWs equally in both study arms, These included individual pictorial checklists developed by the project team for home-monitoring of pregnant/postpartum mothers and infants aged 0–23 months^46^ and for newborns (checklist adapted from the SEARCH Program^48^). Other tools previously developed or adapted and validated were for community referral, supervision and reporting, mothers’ birthing plan to keep at home,^46^ and community development planning.^49^

Trainers, CSs, and CHWs were selected and trained between 2010 and 2014 in either the experimental or control teaching method based on their corresponding PHC facility cluster. Twenty-three selected heath staff from 11 experimental PHC facilities were trained as trainers in the SH teaching methodology, and 23 staff from 11 control PHC facilities were trained separately in standard CHW training methods. Trainers received 14 nonconsecutive days of training: 6 days in adult learning methodologies and 8 days in use of the corresponding set of 8 training manuals by type of teaching methodology (1 for each of the 7 topics plus 1 introductory manual).

Monthly 1-day training workshops for about 500 CHWs and 46 CSs were organized and held by trainers in PHC facilities, unless roads were impassable during the January to March rainy season. Each of the 7 topics was taught in 1 to 3 full-day workshops. Each time the 7 topics were completed, another round of monthly workshops was initiated.

### Study Objective

The study objective was to test the attributable effect of SH on child stunting: height-for-age less than -2 Z-scores below the median according to the WHO growth standard.^50^ The 2 study groups were defined as (1) mothers and children living in PHC facility catchment areas where CHWs were trained with the SH teaching method (experimental clusters); and (2) mothers and children living in catchment areas where CHWs were trained using standard methods (control clusters). Both study groups received home visits by experimentally trained or standard method trained CHWs, respectively.

The study tested the attributable effect of Sharing Histories on child stunting.

### Study Design

We conducted a cluster-randomized controlled trial (cRCT) in 22 clusters, with each cluster comprising a PHC facility and its catchment area population. A cRCT design was appropriate in this study based on criteria to select best methods to evaluate behavior change techniques.^51^ The cRCT design enabled overcoming the difficulties of using distinct training methods for individually randomized CHWs and potential contamination between study groups. This trial is registered with ClinicalTrials.gov, NCT02903602.

All PHC facilities and their catchment areas in 3 municipal districts were included. Clusters were matched in pairs on 2 criteria: category of PHC facility resolutive capacity and distance from district capital. Matched pairs were then randomly allocated to either study arm by the principal investigator. Interventions were applied at the cluster level for health personnel trainers, CHWs, and CSs. Type of CHW training method depended on the cluster to which they had been randomized.

**FIGURE 2.**
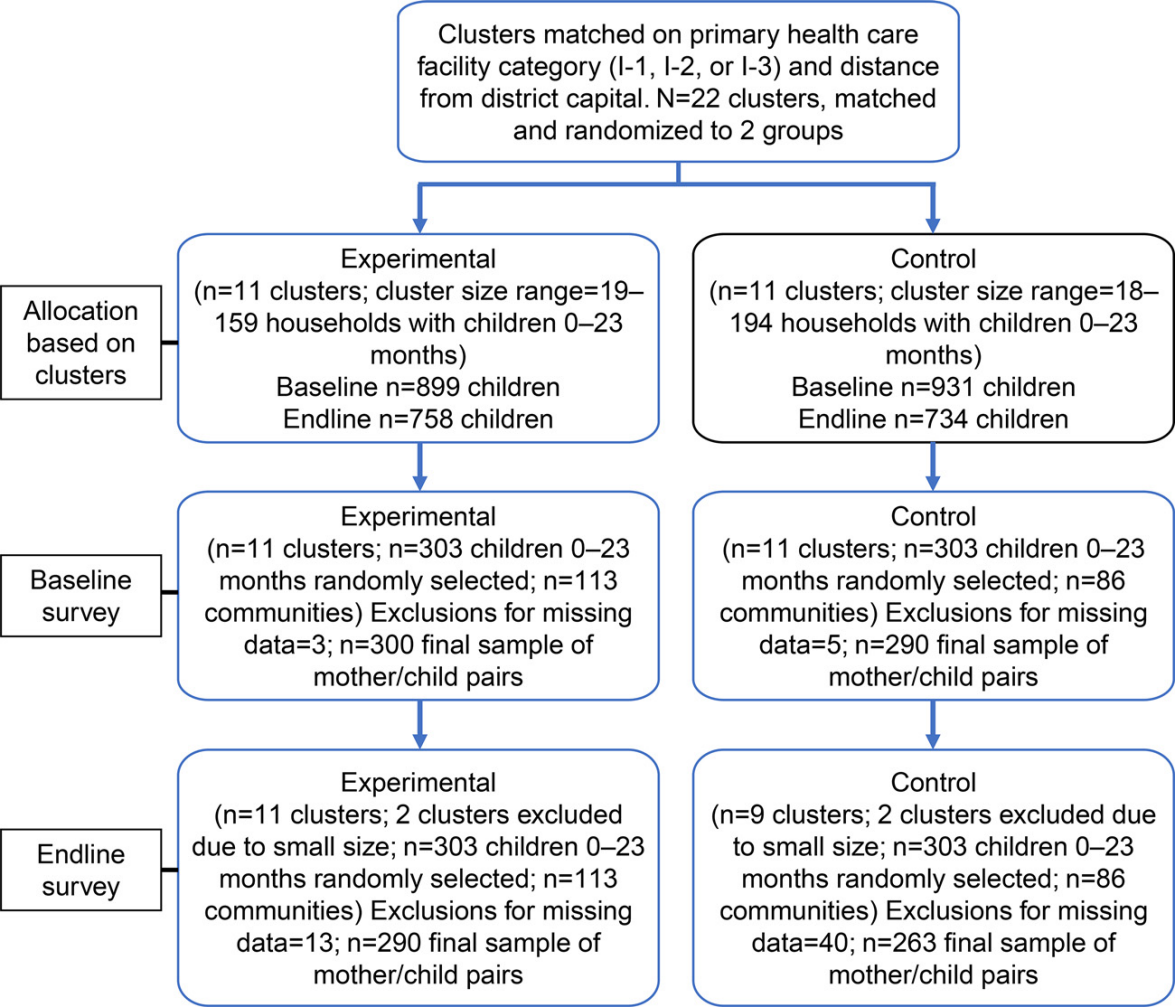
Allocation of Clusters and Study Population, Huánuco Region, Peru

At endline, 2 small communities did not fall into the systematic random sampling of sampling clusters (as distinguished from the randomized intervention clusters), due to the small size of those communities and the low number of children aged 0–23 months. Thus, it was necessary to exclude each of their respectively matched communities with which they had been matched before the baseline survey. As a result, 18 clusters were included in final data analyses. Outcomes were measured in 2 independent samples of households at baseline and endline. The allocation of study clusters is illustrated in Figure 2.

### Blinding

The study was triple-blinded (participants, program implementers, and outcomes assessor). Ministry of Health and municipal officials were unaware of the 2 different teaching methods being used. PHC staff who served as CHW trainers were trained in separately programmed workshops and were unaware of differences in the training methods being used. Experimentally trained CHWs and CSs had no contact with peers from control clusters. Household survey teams and data managers were blinded to the study groups.

### Project Evaluation

Several data collection methods were used to assess baseline, process, and impact. Baseline qualitative studies were conducted on key home practices for maternal and child health and nutrition; CHW efforts in communities; views of community and municipal leaders of health actions; and assessments of PHC facilities in the project area regarding their level of organization for quality services and for work in communities. Project monitoring provided data on CHW training attendance, pre- and posttests of CHW learning in training workshops, and completion of tasks by CSs. At the project’s end, a sample of CHWs and all CSs were interviewed with close-ended questions. An in-depth qualitative study was conducted at endline with a sample of CHWs, CSs, and trainers. Most importantly, repeat household interview surveys were conducted at baseline and endline to evaluate the impact of the experimental training intervention.

### Methods for Household Interview Surveys

Cross-sectional household surveys were conducted at baseline and at 4-year follow-up on 2 independent samples of mothers with children aged 0–23 months in the 2 study areas. The surveys were conducted, under guidance of the study team, by an independent research institution (Instituto de Investigación Nutricional) that was blinded to study groups.

#### Study Variables

The main outcome variable was stunting, height-for-age less than −2 Z-scores^50^, as a proxy measure of health status in children aged 0–23 months. Independent variables previously shown to be associated with child stunting were measured, considering the following constructs: (a) birth weight, (b) breastfeeding practices, (c) child morbidity, (d) early home treatment of child illness, (e) use of health services for prevention and illness, (f) complementary child feeding practices, (g) micronutrient consumption in pregnancy and infancy, (h) WASH practices, and (i) mothers’ knowledge of danger signs during the first 1,000 days. We also asked mothers about their receipt of benefits from selected government health and social services and any CHW home visits during and after their most recent birth.

#### Survey Sampling Frame

We obtained a list of children in each community from registers at each PHC facility. The study area had 22 health facility clusters with approximately 1500 children aged 0–23 months, yielding a sample that represented about 40% of these children.

#### Survey Sample Size

Given a baseline value of 35% stunting in children aged 0–23 months, we expected a reduction from 35% to 22% in the experimental study arm and from 35% to 30% in the control arm. With the number of children of eligible age in the study area limited to 1500, we applied a finite population correction to our sample size calculation.^52^ With a 95% confidence level (CI) and 80% power, each study group was estimated at 283 cases. An assumed 7% nonresponse rate gave a total of 303 cases per study group.

#### Survey Sample Selection

Two-stage sampling of households was conducted independently for experimental and control study arms. In the first stage for each study group, a list of communities and children aged 0–23 months in each randomized study cluster (PHC facility catchment area) was obtained from PHC facility registries. From this list, 38 sampling clusters (as distinguished from study cluster) were randomly selected by a systematic process with a random start. The number of sampling clusters in a community was proportional to its size, and each cluster had 8 children.

The second stage of sampling for household surveys was conducted upon arrival in a selected community. Permission to interview was obtained from village leaders who also helped to identify children born in the previous 4 months, who were then added to the list from the respective health facility registry. Children’s names were alphabetically listed and systematically selected with a random start. This method avoided selecting 2 children from the same family. If a community had fewer than the required number of eligible children, the team moved to the next closest community to reach the needed number.

#### Survey Instrument

The survey questionnaire was adapted from the Demographic and Health Survey instrument to allow direct comparison of results with national and international survey data.^3^ Questions were added to measure exposure to CHW visits and teaching materials, as well as empowerment and WASH indicators.

#### Survey Team

Experienced local field interviewers and anthropometrists were competitively selected and trained for baseline and final surveys. One-third of each team had full or working knowledge of Quechua, the indigenous language. Anthropometrists (nurses) received standardized training for child length and weight measurement and were assisted in the field by the interviewer. Training of survey teams lasted 1 week and focused on the consent process and use of the instrument, with supervised practice with mothers and their young children from outside the study area.

#### Anthropometry Measures

Digital platform scales were used to measure child weights, and their precision and accuracy were checked to 100 g before use. The mother was weighed alone first and then with the child wearing only light clothing. Both weights were recorded for later consistency checking. Length was measured using a lightweight folding durable plastic infant-o-meter accurate to 1 mm,^53^ with a minor modification to prevent movement of the foot board if the child pushed against it.

#### Survey Field Supervision

Supervisors accompanied each fieldworker to check survey forms in the field for completeness, and to conduct periodic repeat surveys with 10 questions after the main interview by the fieldworker.

#### Survey Data Entry and Analysis

Double data entry was done using Visual Fox Pro. Consistency and range checks were also done, and data were checked against the original forms as needed. Breastfeeding and infant feeding practices based on 24-hour dietary recall were evaluated using standard WHO indicators.^54^ Child anthropometry measures were converted to Z-scores of height-for-age, weight-for-age, and weight-for-height, using the 2006 WHO growth standard.^50^

We conducted an intention-to-treat analysis considering all sampled subjects in the study areas. Univariate assessment and bivariate tests of association were conducted on a wide range of independent variables to establish comparability of study groups and to identify potential predictors of child stunting. Bivariate regressions of the outcome stunting on independent variables were assessed with generalized estimating equations (GEE) to adjust for clustering.

We conducted 2 stages of a difference-in-differences (DID) analysis to test the differences from baseline to endline of the experimental intervention on children’s mean height-for-age Z-score (HAZ). We first conducted a standard DID analysis for all children, then by subgroups stratified by low and high maternal literacy. The DID analyses compared mean HAZ by 9 pairs of matched clusters, comparing baseline to endline. Next, we compared 2 levels of maternal literacy within each study group to quantify the effect of the observed interaction of the experimental intervention by stratified levels of high and low maternal literacy on stunting.

Finally, we built a GEE model on endline data to determine the effect of the experimental intervention in interaction with maternal literacy versus the effect of the control intervention on the outcome stunting. Covariates with a *P*-value of .20 or less in the bivariate regressions on stunting were tested for inclusion in the multivariate model. Covariates that were colinear with the outcome variable were not included. Data were analyzed using SPSS version 17 for the baseline and version 20 for the final survey.

#### Ethics and Informed Consent

The Institutional Research Board of the *Instituto de Investigación Nutricional* approved the household survey proposal and consent process. Consent was verbal using an approved standardized protocol.

### Methods for Close-Ended Interviews With CHWs and CSs

Fifty CHWs from each study group (n=100) were interviewed after the intervention period to identify their sociodemographic characteristics and perceptions of the training received, their roles in teaching mothers, their participation in CHW groups with their CS, and changes in themselves as a result of the training. CHWs were randomly selected from a list of active CHWs in each study cluster, proportional to the number of CHWs in each cluster. Interviews were conducted by nursing students from the local university following training and practice in interviewing techniques from expert interviewers. We used a closed-ended questionnaire adapted from Care Group program materials.^55^

All CSs (46) were interviewed following the intervention period for the same reasons as listed for the CHW interviews. In addition, the CS interviews aimed to identify their perceptions of collaboration with health personnel, community leaders, and local government. We adapted a closed-ended questionnaire developed for Care Group programs.^55^

### Methods for In-Depth Qualitative Study

Following the intervention period, to triangulate quantitative findings, individual in-depth interviews were conducted with trainers, CSs, and CHWs from the experimental group on their experiences and opinions regarding SH as a teaching/learning methodology. Control group participants were interviewed on their training and learning experiences. Informed consent was obtained for all interviews. Using a unique interview guide for each type of respondent, interviews were recorded, translated from Quechua to Spanish as needed, and transcribed into Microsoft Word. Analysis was done by a trained medical anthropologist with Atlas.ti software.^56^

## RESULTS

### Results of Intervention Monitoring

Attendance by CHWs and CSs was 82% or better for 5 of 6 workshop topics in the monthly trainings offered at the 22 PHC facilities. Additional small-group training sessions were run by CSs for their respective CHWs once or twice a month within communities for reinforcement of learning. CHWs covered missed workshops during these small group sessions or through a CS visiting them at home to provide personalized training.

Verbally applied knowledge tests were given to CHWs before and after completing each training module topic. Pretest scores of CHWs averaged 40% and improved substantially to about 80% on posttests. Experimentally trained CHW had posttest scores much higher than controls on the growth and nutrition and the diarrhea modules, which were 90% and 96% for the experimental group, respectively; the CHW control group scored 82% and 74%, respectively. CHW workshop attendance and pre-post test scores are reported in the Supplement.

### Baseline and Follow-Up Household Survey Results

#### Comparability of Study Groups on Demographic Characteristics

Mothers were comparable between study groups by age, parity, and education. More control group mothers worked for cash or barter in the follow-up survey compared with experimental group mothers. Children were comparable between study groups in both surveys by age, sex, mean birthweight, and proportion with low birth weight (Table 3).

**TABLE 3.** Demographic Characteristics of Mothers and Children, by Study Group and Survey, Cluster-Randomized Controlled Trial on Sharing Histories CHW Training Methodology, Huánuco Region, Peru

**Demographic** Characteristics	**Baseline Survey 2010**			**Final Survey 2014**		
	**Study Group**		**Significance**	**Study Group**		**Significance**
	**Experimental (n=308)**	**Control (N=298)**		**Experimental (n=290)**	**Control (n=263)**	
Mothers						
Age, years, mean (SD)	26.9 (7.8)	27.2 (9.2)	.62	27.1 (7.8)	26.1 (6.8)	.11
Number of children, mean (SD)	2.6 (1.9)	2.8 (2.2)	.18	2.7 (1.8)	2.6 (1.6)	.25
Distribution of number of children, %			.14			.07
1	37.5	33.0		35.9	32.5	
2	22.6	27.9		22.8	28.5	
3–4	26.2	21.4		22.8	27.0	
5–12	13.6	17.7		18.6	12.2	
Total	100.0	100.0		100.0	100.0	
Education, years, mean (SD)	4.4 (3.7)	4.7 (3.6)	.35	5.4 (3.9)	5.8 (3.5)	.21
Distribution of maternal educational level, % (n)			.60			.37
No education or cannot read (illiterate)	31.5 (97)	32.3 (96)		24.8 (72)	20.2 (53)	
Any primary education (literate)	46.2 (142)	42.5 (127)		40.7 (118)	45.2 (119)	
Any secondary or more (literate)	22.3 (69)	25.2 (75)		34.5 (100)	34.6 (91)	
Total	100 (308)	100 (298)		100 (290)	100 (263)	
Works for cash or barter, %	12.8	17.3	.25	34.1	41.8	.04
Children						
Age in months, mean (SD)	11.4 (6.7)	10.9 (6.8)	.39	11.0 (7.0)	11.3 (6.6)	.58
Sex of child, female, %	47.7	47.3	.49	50.7	47.9	.29
Birth weight (g), mean (SD)	3,045 (471)	3,042 (285)	.92	3,051 (444)	3025 (481)	.52
For illiterate mothers	3,063 (454)	2,953 (494)	.13	2,980 (448)	3076 (475)	.26
For literate mothers	3,042 (479)	3,080 (436)	.41	3,074 (442)	3013 (482)	.17
Low birth weight (<2,500 g)	10.2	7.2	.30	9.8	10.0	.92
For illiterate mothers	10.3	10.2	.98	9.9	8.2	.75
For literate mothers	10.2	6.7	.20	9.8	10.5	.81

Abbreviation: CHW, community health worker; SD, standard deviation.

#### Reported Household Visits by Community Health Workers

As reported in the endline household survey, the proportion of mothers who received 1 or more home visits from a CHW during pregnancy or after birth was 63.1% in experimental clusters and 60.5% in control clusters, with a similar distribution of number of visits by study group. Mothers in the experimental group were more likely to receive 1 or more CHW visits compared with those in the control group among those with any primary school education (72.0% experimental versus 66.4% control, *P*<.01). Among those visited, mothers in both study groups received an average of 5.3 visits from a CHW before and/or after their most recent pregnancy, with a range of 1 to 27 visits. (Table 4).

**TABLE 4.** Home Visits From CHWs Received by Mothers at Endline Survey, 2014, by Study Group, Huánuco Region, Peru

**Receipt by Mothers of CHW Home Visits**	**Experimental (n=290)**	**Control (n=263)**	**Significance**
Mothers who received 1 or more CHW home visits, %	63.1	60.5	.29
Distribution of mothers who received 1 or more home visits by educational level, %			.59
No education or cannot read (illiterate)	23.5	18.9	
Any primary education and can read (literate)	46.4	49.7	
Any secondary or more education (literate)	30.1	31.4	
Total	100.0	100.0	
Mothers who received 1 or more CHW visits received within each educational level, %			
No education or cannot read (illiterate)	59.7	56.6	.43
Any primary education and can read (literate)	72.0	66.4	.21
Any secondary or more education (literate)	55.0	54.9	.55
Number of CHW home visits received (range 1–27), mean (SD)	5.28 (4.6) (N=180)	5.27 (4.3) (N=159)	.99
Number of CHW home visits received within each educational level (range 0–27), mean (SD)	3.28 (4.5)	3.19 (4.2)	.81
No education or cannot read (illiterate) (range 0–27)	2.65 (3.9)	2.98 (4.1)	.65
Any primary education and can read (literate) (range 0–27)	3.51 (4.0)	3.46 (4.3)	.93
Any secondary or more education (literate) (range 0–27)	3.45 (5.3)	2.94 (4.2)	.47
Distribution of number of CHW visits received by mothers, %			.88
0	37.9	39.2	
1 or 2	19.3	19.0	
3–5	22.4	20.2	
6–10	13.1	15.2	
11–27	7.2	6.1	
Total	100.0	100.0	

Abbreviations: CHW, community health worker, SD, standard deviation.

Among those visited, mothers in both study arms received an average of 5.3 visits from a CHW before and/or after their most recent pregnancy.

#### Changes in Knowledge and Practices of Study Mothers

**Knowledge of Key Danger Signs.** Maternal spontaneous knowledge of 2 or more danger signs on each of 4 topics (pregnancy, birth, postpartum, and newborns) increased significantly in both study groups from baseline to endline. Improvements from baseline to endline were greater in the experimental group than in the control group regarding danger signs in pregnancy and postpartum. The control group of mothers had greater improvement in knowledge of danger signs in newborns (Table 5).

**TABLE 5.** Changes in Maternal Knowledge and Practice, by Study Group, Huánuco Region, Peru

		**Baseline Survey 2010**			**Endline Survey 2014**			**Baseline to Endline Differences**	
		**Experimental N=308**	**Control N=298**	**Significance**	**Experimental N=290**	**Control N=263**	**Significance**	**Experimental**	**Control**
Proportion of mothers with spontaneous correct report of at least 2 danger signs, %									
Danger signs in pregnancy		39.3	42.6	.28	73.8	74.5	.46	+34.5	+27.9
Danger signs during birth		11.0	7.7	.10	27.6	24.7	.25	+16.6	+17.0
Danger signs in postpartum		20.5	23.2	.24	38.3	35.7	.30	+17.8	+12.5
Danger signs in newborns		23.1	18.1	.08	61.0	61.6	.48	+37.9	+43.5
Micronutrient consumption, %									
Mothers consuming iron tabs 3+ months last pregnancy		51.0	46.6	.16	77.2	74.1	.41	+26.2	+27.5
Children with micronutrients added to food in past 24 hours		0.0	0.3	.49	57.9	61.2	.24	+57.9	+60.9
Children with vitamin A supplement in past 6 months		47.1	46.3	.77	21.3	19.2	.28	−25.8	−27.1
Proportion of children with nutritional pattern, %									
Currently breastfeeding		89.6	87.2	.36	88.3	90.1	.29	−1.3	+2.9
Early breastfeeding within 1 hour of birth		77.6	72.8	.11	69.0	66.5	.30	−8.6	−6.3
Exclusive breastfeeding, 0–5 months		71.8 (N=71)	83.5 (N=79 )	.06	86.6 (N=82)	90.2 (N=61)	.35	+14.8	+6.7
Food consumption in past 24 hours for children aged 6–23 months, %		(N=237)	(N=219)		(N=208)	(N=202)			
Iron-rich foods		53.2	53.9	.48	93.8	93.1	.47	+40.6	+39.2
Animal protein		32.5	37.4	.16	47.1	49.0	.39	+14.6	+11.6
Minimum meal frequency		69.6	68.0	.40	94.2	89.6	.06	+24.6	+21.6
Minimum food diversity		56.1	58.4	.34	79.3	75.7	.23	+19.6	+17.3
Household water, sanitation, and hygiene practices by maternal literacy, %									
No animals (except pets) live inside house	Illiterate	45.0	50.0	.31	52.8	64.2	.14	+7.8	+14.2
	Literate	50.6	56.9	.14	65.6	58.6	.08	+15.0	+1.7
Uses correct treatment for drinking water	Illiterate	80.2	89.5	.17	97.2	79.2	.00	+17.0	−10.3
	Literate	86.1	85.9	.29	92.2	96.2	.06	+6.1	+10.3
Mother washes hands after defecating	Illiterate	28.1	29.5	.48	23.6	34.0	.14	−4.5	+4.5
	Literate	50.7	43.2	.08	50.5	39.0	.01	−0.2	−4.2
Soap is available for hand washing^a^	Illiterate	na	na	—	48.6	37.7	.15	—	—
	Literate	na	na	—	61.5	61.0	.50	—	—
Uses safe water source	Illiterate	49.0	43.2	.26	66.7	63.5	.43	+17.7	+20.3
	Literate	64.1	59.8	.21	73.0	73.1	.54	+8.9	+13.3
Improved cook stove installed in past 4 years	Illiterate	51.0	60.0	.14	40.3	22.6	.03	−10.7	−37.4
	Literate	50.2	43.7	.11	40.4	32.9	.07	−9.8	−10.8
Does not use wood or dried dung as cook fuel	Illiterate	6.3	2.1	.14	11.1	7.5	.36	+4.8	+5.4
	Literate	18.7	15.1	.20	30.7	26.2	.18	+12.0	+11.1
Receipt of government health and social services, %									
Infant food supplementation program		93.8	92.3	*.28*	0.7	1.1	*.45*	−93.1	−91.2
Conditional cash transfer program (Juntos)		52.6	53.0	*.49*	53.8	52.9	*.45*	+1.2	−0.1
Municipal Glass of Milk program		76.0	76.2	*.52*	68.6	68.1	*.48*	−7.4	−8.1
Child antiparasite treatment in past 6 months		12.1	15.4	*.13*	9.0	6.1	*.13*	−3.1	−9.3
Participation of mothers in women’s groups with discussion of child health and nutrition^a^		46.4	44.3	*.60*	50.0	49.4	*.48*	+3.6	+5.1

a Not assessed at baseline.

**Micronutrient Consumption.** Study mothers increased their consumption of iron tablets for the standard minimum of 3 or more months during pregnancy at an approximate rate of 50% increase in both study groups. Study children consumed micronutrient powder (Sprinkles) added to food in the previous 24 hours in approximately 60% of both study groups at endline. No micronutrients were taken at baseline by either group (Table 5).

**Key Breastfeeding Practices.** Nearly 90% of all study children were breastfeeding at the time of both surveys. At baseline, three-fourths of study cases had initiated breastfeeding within 1 hour of birth, but this declined to two-thirds in both groups at endline. Exclusive breastfeeding in children 0–5 months of age increased significantly from baseline to endline by 14.9 points in the experimental group (71.8% to 86.7%) (χ^2^(1,N=602)= 20.4; *P*<.01), but by only 6.7 points in the control group (83.5% to 90.2%) (χ^2^(1,N=598)=5.6; *P*<.05) (Table 5).

**Key Child Feeding Practices.** Children’s consumption of iron-rich foods notably increased in both study groups from 53% at baseline to 93% at endline. Animal protein consumption by children also increased significantly in both study groups but reached less than 50% at endline (Table 5). Minimum meal frequency, established as 3 meals per day on average according to age and breastfeeding status,^54^ was 2.2 meals per day for infants 6–11 months at baseline increasing to 3.5 per day at endline. The experimental group had a significantly higher percentage of minimum meal frequency than controls at endline (94.2% experimental versus 89.6% control) (χ^2^(1, N=584)=4.1; *P*<.05) (Table 5). Minimum dietary diversity by breastfeeding status^54^ showed significant increases in both study groups but differences between study groups were nonsignificant at endline (Table 5).

**Key Water, Sanitation, and Hygiene Practices.** WASH practices at baseline were generally much better among literate mothers than nonliterate mothers in both study groups. At endline, several key WASH practices differed significantly within strata of maternal literacy. Among literate mothers, no animals living inside the home (except pets) and mother washing hands after defecation were significantly more frequent in the experimental group compared to the control group. Among illiterate mothers, correct treatment of drinking water and installation of an improved cook stove in the past 4 years were significantly more frequent in the experimental versus control group (Table 5).

Among literate mothers, handwashing after defecation and not raising animals inside the home were more frequent in the experimental group at endline.

**Government Health and Social Services Received by Mothers and Children.** Study groups were comparable on receipt of government services for mothers and children that might affect child growth. Receipt of a government food supplement (instant fortified weaning food) by children aged 6–23 months was nearly universal at baseline but null at endline because the food program was discontinued in 2012. One-half of all study mothers received $30 per month as a conditional cash transfer (Juntos Program). The municipal Glass of Milk program provided a daily milk ration to about two-thirds of all mothers with a child 0–5 months of age and to children aged 6–23 months. Government distribution of antiparasitic medication and vitamin A supplements declined during the project period. One-half of all mothers participated in a women’s group in which health and nutrition topics were discussed (Table 5).

### Baseline to Endline Changes in Child Stunting

#### Changes in Stunting by Demographic Characteristics

The baseline prevalence of child stunting was 34%–35% in both study groups, unadjusted for clustering. Stunting was reduced in experimental clusters by 4.1% from baseline (34.4%) to endline (30.3%), while stunting in control clusters plateaued from baseline (35.3%) to endline (35.0%) (Table 6). The difference at endline is not significant.

**TABLE 6.** Changes in Prevalence of Growth Stunting in Children Aged 0–23 Months by Demographic Characteristics, Study Group, and Survey, Huánuco Region, Peru

	**Baseline Survey 2010**			**Endline Survey 2014**		
	**Experimental (N=305)**	**Control (N=295)**	**Significance**	**Experimental (N=290)**	**Control (N=263)**	**Significance**
All study children	34.4	35.3	.45	30.3	35.0	.14
Mother’s educational level						
No education or cannot read	45.3	43.6	.47	47.2	39.6	.25
Any primary or secondary	29.0	32.0	.29	24.8	33.8	.03
Child’s age						
0–11 months	22.9	22.7	.53	19.1	25.9	.10
12–23 months	46.6	48.3	.43	43.6	45.2	.45
Child’s sex						
Female	21.9	28.6	.12	22.4	29.4	.12
Male	45.9	41.3	.24	38.5	30.1	.43
Child birth weight						
≤2,500 g	44.8	61.9	.18	50.0	46.2	.49
>2,500 g	32.6	33.0	.50	27.9	33.5	.11

Stunting was reduced in experimental clusters by 4.1% from baseline to endline, while stunting in control clusters plateaued from baseline to endline.

At both baseline and endline, stunting was much lower in children of literate mothers compared with children of illiterate mothers, in children aged 0–11 months compared with those aged 12–23 months, in girls compared with boys, and in those with normal birthweight (2,500 g or more) compared with those with low birth weight (≤2,500 g) (Table 6).

At endline, stunting prevalence among children of literate mothers with any primary or secondary education was significantly lower in the experimental group at 24.8% versus 33.8% among controls. Among children with normal birth weight >2,500 g, stunting was lower in the experimental group (27.9%) than in the control group (33.5%) (Table 6).

In accordance with the findings of significantly greater reduction in stunting in children of literate mothers in the experimental group, we found that these mothers, compared with control group peers, had reported the following more frequently: no animals kept within the home (65.5% experimental versus 58.6% control, *P*<.05); handwashing after defecation (50.5% experimental versus 39.0% control, *P*<.05); and provision of a minimum number of feeds per day to their child (94.2% experimental versus 89.6% control, *P*<.05) (Table 5).

##### Changes in Stunting in the per Protocol Subgroup

Mothers who received 1 or more CHW visits were significantly less educated than mothers who reported no visits. Nevertheless, the prevalence of child stunting did not differ between the visited and non-visited groups (Table 7).

**TABLE 7.** Changes in Prevalence of Growth Stunting in Children Aged 0–23 Months by Receipt of 1 or More CHW Visits, by Maternal Literacy and Study Group at Endline, Huánuco Region, Peru

	**Both Study Groups**		**Significance**	**With CHW Visit**			**Without CHW Visit**		
	**With CHW** **Visit**	Without CHW Visit		**Experimental**	**Control**	**Significance**	**Experimental**	**Control**	**Significance**
All mothers									
Child stunting, %	33.3	31.3	.34	29.0	38.4	.04	37.2	29.8	.38
Maternal education in years, mean (SD)	5.3 (3.5)	6.0 (3.9)	.02	5.1 (3.6)	5.6 (3.4)	.20	5.9 (4.2)	6.1 (3.7)	.71
N	342	211		183	159		107	104	
Illiterate									
Child stunting, %	47.9	38.5	.19	48.8	46.7	.52	44.8	30.4	.22
Maternal education in years, mean (SD)	0.7 (1.3)	1.2 (1.4)	.08	0.7 (1.3)	0.8 (1.2)	.67	1.0 (1.4)	1.4 (1.4)	.38
N	73	52		43	30		29	23	
Literate									
Child stunting, %	29.4	28.9	.51	22.9	36.4	.01	28.2	29.6	.49
Maternal education in years, mean (SD)	6.5 (2.9)	7.6 (3.1)	.00	6.4 (3.0)	6.7 (2.7)	.49	7.8 (3.3)	7.5 (2.9)	.58
N	269	159		140	129		78	81	

Abbreviations: CHW, community health worker, SD, standard deviation.

**FIGURE 3.**
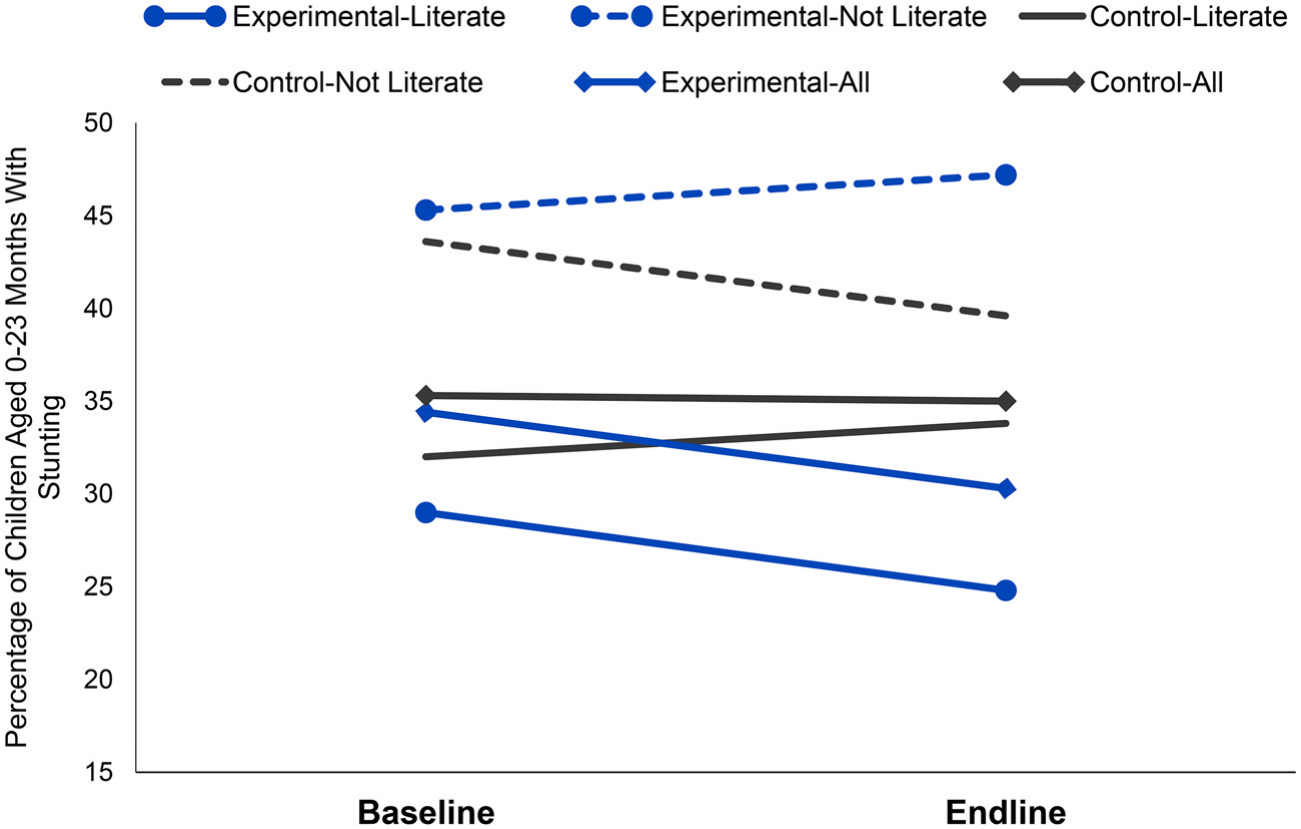
Changes in Stunting in Children Aged 0–23 Months Before and After a Community Health Worker Training Intervention Comparing Experimental and Control Groups by Maternal Literacy, Huánuco Region, Peru

In the per protocol analysis of mothers who had been visited by CHWs, stunting was present in 29.0% of children of mothers visited by an experimentally trained CHW compared with 38.4% in children of mothers served by control CHWs (*P*=.04). Among literate mothers, these percentages were 22.9% and 36.4%, respectively, for experimental and control groups (*P*=.01) (Table 7 and Figure 3). Literate mothers were 78% of the study population at endline.

### Predictors of Child Stunting

Bivariate regressions of potential predictors of child stunting, including type of CHW learning method (SH versus control) and potential covariates, are shown in Table 8, adjusted for clustering. The experimental CHW learning method and maternal literacy were found to interact in relation to stunting. That is, learning method was associated with a decrease in stunting among children of literate mothers, but not among children of illiterate mothers. The interaction term had a significant independent association with child stunting (Beta=0.75; 95% confidence interval=0.20, 1.30; *P*<.00) (Table 8). Covariates that had bivariate association with stunting significant at *P*<.20 included child age in months, sex of child, low birth weight (<2,500 g), birth weight in grams, child consumed the minimum diversity of food in the previous 24 hours, number of months that mother took iron tablets during pregnancy, child consumed multi-micronutrient + iron supplement in the past 24 hours, household has an improved cookstove installed in the previous 4 years, and child had parasite treatment in the past 6 months. Variables that were colinear with the outcome (birth weight in grams, child age in months) were not included in the multivariate model.

**TABLE 8.** Generalized Estimating Equations^a^ Bivariate Associations With Child Stunting for 553 Children Aged 0–23 Months, Huánuco Region, Peru

Predictors of Child Stunting^b^	Estimate Beta	95% Confidence Interval	Significance
Experimental group	0.21	−0.31, 0.74	.43
Mother is literate	0.64	0.30, 0.98	<.00
Interaction: experimental group × maternal literacy	0.75	0.20, 1.30	<.00
Maternal characteristics			
No. of children born in mother’s lifetime (1–12)	−0.12	−0.24, 0.01	.07
Mother has remunerated work (0, 1)	0.00	−0.34, 0.34	.99
Child characteristics			
Age (0–23 months)	−0.08	−0.10, −0.05	<.00
Female child (0, 1)	0.63	0.26, 0.99	<.00
Low birth weight (<2,500 g)	−0.75	1.32, −0.72	.01
Birth weight (g)	.00	.00, .00	<.00
Child feeding variables			
Receives breastmilk within 1 hour of birth (0, 1)	−0.19	−0.62, 0.24	.39
Meets minimum food diversity (0, 1)	−0.57	−1.18, .054	.07
Meets minimum meal frequency (0, 1)	−0.17	−0.86, 0.53	.64
Consumes iron-rich foods in past 24 hours (0, 1)	−0.35	−1.47, 0.78	.55
Consumes animal food source past 24 hours (0, 1)	0.02	−0.55, 0.60	.94
Micronutrient consumption			
Mother took iron during last pregnancy (0–9 months)	0.09	−0.02, 0.17	.06
Child consumed Sprinkles^c^ in past 24 hours (0, 1)	−0.45	−0.86, −0.04	.03
Vitamin A capsule taken by child in past 6 months (0, 1)	−0.04	−0.56, 0.49	.89
Water, sanitation, and hygiene practices			
Mother washes hands after defecation (0, 1)	0.10	−0.17, 0.36	.46
Soap, ash, or detergent used to wash hands (0, 1)	0.03	−0.19, 0.24	.80
Household has safe water source (0, 1)	−0.35	−0.85, 0.15	.17
Drinking water is treated correctly (0, 1)	0.20	−0.33, 0.73	.45
Improved cook stove installed in past 4 years (0, 1)	−0.44	−0.85, −0.03	.03
Non-pet animals do not live in the home (0, 1)	0.14	−0.26, 0.54	.50
Government health and social services			
Parasite treatment for child in past 6 months (0, 1)	−0.90	−1.43, −0.37	<.01
Mother in participatory women’s group (0, 1)	−0.08	−0.47, 0.31	.68
Glass of Milk daily ration for child (0, 1)	−0.08	−0.48, 0.33	.72
Juntos cash transfer received by mother (0, 1)	−0.10	−0.46, 0.27	.59

Abbreviation: CHW, community health worker.

a Adjusted for clustering.

b Outcome variable: stunted=1, not stunted=0.

c Multi-micronutrients with iron.

The experimental CHW learning method and maternal literacy were found to interact in relation to stunting.

### Effect of the Experimental Training Intervention on Stunting

#### DID Analysis

We conducted a DID analysis for all children, and for stratified subgroups of maternal literacy. Results in Table 9 show that for all children, the mean HAZ change was not significantly different between experimental and control clusters (*P*= .469). However, in the subgroup of literate mothers, the mean HAZ improved by an average of 1.03 points on the Z-scale in experimental clusters as compared to control clusters between baseline and endline, with a significance level of *P*=.059. For the subgroup of illiterate mothers, the changes between baseline and endline for experimental versus control clusters were not significantly different.

**TABLE 9.** DID Analysis of Mean HAZ in 533 Children Aged 0–23 Months, Baseline to Endline, for All Children and for Subgroups of Children Stratified by Maternal Literacy, Huánuco Region, Peru

Group	Control			Experimental			Paired t-test		
	MeanHAZ Baseline,Mean (SD)	MeanHAZEndline,Mean (SD)	Diff. of Means, Mean (SD)	MeanHAZ Baseline,Mean (SD)	MeanHAZEndline,Mean (SD)	Diff. of Means, Mean (SD)	Paired DID, Mean (SD)	T-Statistic(df)	*P* Value
All children	−1.55 (.46)	−1.55 (.66)	−0.0002 (0.36)	−1.62 (0.47)	−1.47 (0.31)	0.15 (0.46)	0.15 (0.60)	0.760 (8)	.469
Stratified by literacy of mother									
Illiterate	−1.79 (0.51)	−1.57 (0.93)	0.22 (0.59)	−1.77 (0.44)	−1.92 (0.44)	−0.16 (0.57)	−0.38 (0.72)	−1.561 (8)	.157
Literate	−0.80 (1.13)	−1.53 (0.59)	−0.74 (1.01)	−1.60 (0.54)	−1.31 (0.28)	0.29 (0.50)	1.03 (1.40)	2.202 (8)	.059

Abbreviations: df, degrees of freedom; DID, difference-in-differences; HAZ, height-for-age-Z-scores, SD, standard deviation.

We then used DID to test whether the intervention’s effect differed by maternal literacy as an interaction effect. We found that the difference of the mean HAZ from baseline to endline in each of the matched clusters between children of high- and low-literacy mothers in the experimental versus the control group was significant at *P*=.003, using a paired t-test. This finding demonstrated a significant interaction effect of the experimental training method on HAZ by level of maternal literacy.

#### GEE Analysis

Table 10 shows the main variables submitted to the multivariate model using GEE with adjustment for clustering: inclusion in the experimental group, maternal literacy, and the interaction term (experimental group × high maternal literacy) plus covariates. Multivariate results showed that the interaction term had a significant association with stunting (B=.77; 95% CI=0.23, 1.31; *P*< .00). In other words, SH was significantly associated with reduced stunting among literate mothers but not among illiterate mothers. This effect was adjusted by significant covariates associated with stunting: child parasite treatment in the past 6 months, child consumed multi-micronutrients with iron in the past 24 hours, and improved cook stove installed in the past 4 years. Safe water source had a borderline association with stunting.

**TABLE 10.** Generalized Estimating Equations^a^ Multivariate Model for Predictors of Child Stunting With 553 Children Aged 0–23 Months, Huánuco Region, Peru

Predictors of Child Stunting^b^	**Estimates**		**95% Wald Confidence Interval**		**Hypothesis Test**		
	**Beta**	**SE**	**Lower**	**Upper**	Wald Chi-square	**df**	**Significance**
Community health worker training intervention							
Experimental group (0, 1)	−0.27	0.36	0.97	0.43	.58	1	.45
Mother is literate (0, 1)	0.22	0.11	0.00	0.44	3.69	1	.06
Interaction: experimental group × maternal literacy	0.77	0.27	0.23	1.31	7.91	1	<.00
Child nutrition							
Child consumed Sprinkles^c^ past 24 hours (0, 1)	−0.41	0.21	−0.81	.00	3.85	1	.05
Water, sanitation, and hygiene							
Safe water source (0, 1)	−0.43	0.25	−0.92	0.07	2.87	1	.09
Improved cook stove installed past 4 years (0, 1)	−0.49	0.21	−0.91	−0.07	5.26	1	.02
Government health and social services							
Parasite treatment for child in past 6 months (0, 1)	−.85	.34	−1.52	−0.18	6.23	1	.01
Intercept	1.22	0.35	0.54	1.91	12.19	1	<.00
Goodness of fit^d^ with corrected quasi-likelihood under independence model criterion (QICC)	672.97						

Abbreviations: df, degrees of freedom; SE, standard error.

a Adjusted for clustering.

b Outcome variable: stunted=1, not stunted=0.

c Multi-micronutrients with iron.

d Information criteria are in smaller-is-better form.

### Results of Close-Ended Interviews With CHWs and CSs

Results of interviews with 50 randomly selected CHWs from each study group (n=100) and all 27 CSs at project end showed that experimentally trained CHWs and CSs felt more positive about their training and learning than those trained with the control method.

Interviews revealed that experimentally trained CHWs and CSs felt more positive about their training and learning than those trained with the control method.

Experimental CHWs felt more capable of identifying danger signs in mothers and children during the first 1,000 days than control CHWs (38% experimental versus 20% control, *P*<.05).

When CHWs were asked about activities they liked best or found most interesting in the workshops, experimental CHWs liked all the training activities more frequently than control CHWs. Training activities liked best were the use of the SH method (86% experimental versus 60% control, *P*<.01), the pretest (74% experimental versus 52% control, *P*<.05), and the posttest (78% experimental versus 48% control, *P*<.01). Compared with control CHWs, experimental CHWs more frequently liked or found interesting the explanation of the flip charts (92% experimental versus 82% control) and use of participatory dynamics for evaluation of learning at the end of each learning session (86% experimental versus 72% control).

When asked what they had found least entertaining or interesting in the training workshops, control CHWs were more likely to mention disliking all the activities than the experimental CHWs: Sharing Histories (0% experimental versus 8% control, *P*<.05); pretest (14% experimental versus 38% control, *P*<.01); posttest (6% experimental versus 16% control, *P*=.11); explanation of the flip chart (2% experimental versus 12% control, *P*<.05); and participatory dynamics for evaluation (12% experimental versus 26% control, *P*=.07).

Among 27 CSs interviewed, decentralized group meetings held by experimentally trained CSs with their assigned CHWs were more likely to last over 2 hours, compared with meetings held by control CSs (64% experimental versus 31% control, *P*<.05)

In addition, more experimentally trained CSs reported that community leaders supported the work of CHWs in communities than did control CSs, in the following ways: leaders reached out to the municipality to implement actions for women and children (79% experimental versus 69% control); leaders prepared community work plans with activities to protect women and children (71% experimental versus 58% control); leaders gave orders or made community resolutions to encourage families to adopt health practices (36% experimental versus 7% control); and leaders helped women to seek care in primary care facilities or hospitals (29% experimental versus 15% control).

Community leaders in experimental clusters were more likely to be reported as supportive of CHW work than were those of communities in control clusters.

### Results of Qualitative In-Depth Interviews

Postintervention qualitative interviews of CHWs, CSs, and trainers regarding use of SH suggested mechanisms through which the method could have promoted health behavior change first in female CHWs and then in mothers taught by trained CHWs.

To begin with, SH seemed to echo traditional conceptions in this population that knowledge is based on experience.

CHWs noted that after listening to each other’s histories during a training session, seeing a belief or practice listed on paper marked in red for “possibly dangerous effect on health” motivated them to learn why it could be dangerous. After discussing the traditional practice, they discussed what the healthy behavior should be instead, with picture cards (flip chart) later serving to reinforce what the new behaviors should be. (Names of persons cited have been changed to protect privacy.)

*They had us remember how we used to do things and then they taught us.* —CHW Jenny

CHWs’ memories of past experiences were frequently similar because of shared cultural practices. Certain practices that could be harmful, such as home birthing, were often collective practice. Once the practice was mentioned, the group could discuss it openly without recrimination, with a shared acceptance of new learning by CHWs. Hearing about their peers’ differences in practice also provided CHWs with motivation to learn.

*One [CHW] knows…and takes the mother to the hospital, and others [CHWs] don’t know. I felt a greater urge to want to learn*.— CHW Maria

Some memories were emotionally charged, such as pregnancy or birth complications or the death of a child. When strong emotions and tears were expressed, empathetic responses from fellow learners worked to strengthen interpersonal relationships.

*…Some were born badly, sick…. I thought, I thank God because my daughter was born well. On the other hand, there are mothers who suffered. I thought, how is that possible. Sometimes you don’t know.* —CHW Diana

By sharing histories, CHWs seemed to become self-motivated to avoid repetition of their own prior erroneous behavior. CHWs said they felt more confident in their ability to explain new practices to other mothers through the lens of local belief systems; they were able to share newfound knowledge with greater force of conviction.

*It is a good lesson. Because sharing histories you can find out…how that person is living. And you can help them. …You give them confidence and they tell you and a solution can be found. Because by not telling your problems, you don’t find out anything about anybody. But if they tell you their histories, their problems, yes. And you can help them.* —CHW Jenny

CHWs spontaneously shared their own experiences with the mothers under their care, and in turn asked them to share their experiences, thereby promoting empathic connections to strengthen the social relationship, bonding, and trust needed for influencing traditional knowledge.

*I feel better because now I know…what it is to teach mothers. Before I didn’t know. So, on that side I feel good and I have learned to teach.* —CHW Diana

Health personnel who learned to train CHWs with the SH method initially struggled with the new way of training, but soon learned to appreciate the method.

*At first it was difficult but soon it seemed natural. By sharing histories, they take interest in the topic and the new knowledge sticks with them. For us it is easier to teach them like that.* —Trainer María

Some examples of cultural beliefs that were expressed by CHWs during the history-sharing sessions are provided in Table 11.

**TABLE 11.** Examples of Local Beliefs Expressed by CHWs Through Sharing Histories, Huánuco Region, Peru

Topic	**Local Culturally Determined Knowledge, Beliefs, and/or Solutions Identified From CHWs Through Sharing Histories**	**Standard Messages Taught to CHWs by PHC Staff Without Sharing Histories**	Messages Given by PHC Staff Who Are Trained to Facilitate Sharing Histories and Learning With Picture Cards
Pregnancy	Danger signs in pregnancy were not recognized as such. For example, female CHWs did not know that a mother could die if she is bleeding during pregnancy and may consider such bleeding as “normal.” Did not identify pain and frequency of urination as a problem. Heavy work or lifting is continued as normal. Pregnant women eat less to have smaller baby and easier birth. Many foods are specifically avoided during pregnancy, such as fish which may “impede healing.”	Danger signs not generally taught in a way to ensure understanding.	Picture cards are discussed with motivational stories of pregnant women with danger signs and how they can end in death, or how they can end well if care is sought. Mothers should spare their energy by working less and eating more so the infant can have more energy. Picture cards with various images of danger signs are discussed with indications to seek care.
Birth	Institutional birth is not considered desirable due to fears of male health providers and horizontal birth. Women are terrorized by the idea of an episiotomy or cesarean delivery that requires transfer to a hospital distant from home and family. Care by a traditional midwife in the presence of family members is valued. Distance is a major barrier at night and holidays when no means of transport are available.	Home births are illegal. Institutional births are obligatory.	CHWs need to help mothers seek institutional birth with support from family and community members for transport.
Newborns	Newborn danger signs that were recognized as potentially fatal were infant not wanting to eat and infant being flaccid or agitated. Danger signs that were not recognized as such included an odorous umbilical stump. Newborn is placed to one side to first attend the mother immediately after a home birth, sometimes uncovered due to simple negligence.	Information on birth and newborns is not discussed with CHW or mothers: CHWs and mothers “don’t need to know”. Only professional birth and checkups are allowed.	Need for immediate drying and wrapping of newborn and placement with mother for warmth and immediate suckling at the breast. No bath the first day to stay warm. Picture card images of danger signs are provided and discussed with indications to seek care.
Breastfeeding	Insufficient breastmilk is a family trait, so a mother will expect it and nothing can be done if female family members had little milk. Breastmilk is withheld to avoid harming the infant if mother is angry, ill, or is pregnant again. Herbal tea is given frequently (for colic and infant thirst). Dozens of myths surrounding breastfeeding practice are expressed.	Generally, PHC staff are not trained in local breastfeeding beliefs and practice or in correct breastfeeding techniques. Infants are taken away for immediate newborn care and not returned quickly to the mother. Free formula samples are handed out.	All mothers can breastfeed if measures are taken to stimulate milk supply. Herbal tea should not be given to infants, rather the mother should drink the tea. Trainers detect local myths through CHW shared histories and use those to discuss how to avoid insufficient breastmilk and maintain exclusive breastfeeding for 6 months.
Complementary feeding	When a child aged over 6 months does not want to eat, mothers give only breast milk. Mothers value giving liquid soups and semiliquid foods to infants (over semisolids). Animal-source foods are acceptable to give but are not available.	PHC staff recommend taking child off the breast and only give solid food. If breast milk then dries up, give milk formula or cow’s milk.	Continue breastfeeding and try giving small amounts of mashed food more frequently during the day. Soups are mostly water, which fills the infant’s stomach and does not allow space for the food they need to grow. Add citrus juice to legumes to make them more nutritious (increase iron bioavailability) but animal-source foods should be given as much as possible.
Diarrhea	Diarrhea occurs when someone looks at the child with an “evil eye.” When diapers are damp from being hung out to dry overnight, the dampness in the diaper can “enter” the child and cause diarrhea. Traditional healers “pass a cuy (guinea pig)” or “pass an egg” over the child’s body to draw out bad energy. Dirt or lack of hygiene is not associated with diarrhea.	PHC staff promote use of oral rehydration fluid and care-seeking for diarrhea. Use hygiene for prevention (without discussion of local beliefs on causation).	Dirt on hands or on prepared food can cause diarrhea in some cases, aside from other believed causes. Thus, it is best to use hygiene practices to avoid such cases (i.e., handwashing, keep animals out of the home, keep the child off the ground or dirty floor, use correct treatment for drinking water, others).

Abbreviations: CHW, community health worker; PHC, primary health care.

## DISCUSSION

Peru is facing one of the fastest-growing equity gaps among LMICs in the distribution of the benefits of development. The isolated and mostly rural Huánuco Region on the eastern slope of the Andes mountains has one of the highest rates of child stunting (low height-for-age) in the country. Stunting reflects a child’s overall well-being. It accurately indicates social inequalities, which are the cumulative result of poor fetal growth, inadequate nutrition, and infectious disease in the first 2 years of life, associated with deficient home practices for maternal nutrition, breastfeeding, complementary feeding and micronutrient consumption, poor access to health services, and possibly also WASH practices.^57^

Strikingly absent in Peru and elsewhere is a system to reach vulnerable lesser-educated mothers with communication strategies that effectively change health knowledge and practices that affect maternal and child health. In the current study, we tested the effect of Sharing Histories (SH), an innovative CHW teaching methodology that is intended to improve CHWs’ cultural competencies to support improved maternal health behaviors.^38^

Culturally traditional women, even those with primary or secondary education, generally have special needs for effective learning. Through sharing their own experiences and hearing those of other women, CHWs-in-training become quickly attuned to a topic and become interested in hearing about practical solutions to use in specific circumstances, so they know what to suggest to prevent or solve problems. When CHWs take ownership of their past experiences and improve their abilities to express themselves, their self-esteem, self-confidence, and empowerment increase, making them more convincing and effective change agents for health behaviors and for mobilization of appropriate demand for health services.

When CHWs take ownership of their past experiences and improve their abilities to express themselves, their self-esteem, self-confidence, and empowerment increase.

Many countries have attempted to develop links between PHC services, community-based health care resources, and households through work with CHWs. These programs provide a channel for reaching families with information, resources, and referrals. CHWs are particularly well positioned to address health behaviors in a culturally appropriate way, but a challenge is how to help CHWs develop the cultural and personal competencies to support change in other women’s health belief systems. Government PHC staff who are CHW trainers and who use the SH method can learn firsthand the cultural practices of an area to inform their training, without having to rely on costly or nonexistent in-depth research on local practices.

The CHW program described here can be implemented and fully managed at a cost of about US$1.80 per capita per year that can be divided between the health budget (US$1.20) and local government (US$0.60) (see Supplement for cost data).

We hypothesized that the experimental CHW training method could lead to maternal behavior change and subsequent improvement in child growth. This hypothesis was supported by prior research showing that recall of autobiographical memory changes future behavior.^38^ ^,^ ^41^ ^,^ ^58^ Prior supportive research also showed that narrative communication using firsthand and secondhand stories is an effective health communication strategy for health behavior change that subsequently improves health status.^59^

Qualitative findings helped to explain how change occurred. The acceptance by CHWs of the SH training method could be related to the fact that experience is the traditional basis of learning. Women’s personal experiences with childbearing and child rearing are generally not valued, but SH specifically recognizes and builds on them. Rural female CHWs are often shy and feel inhibited speaking in front of people they do not know well. The SH method provides a platform for CHWs to speak aloud in a group and practice public speaking. In standard CHW training, classes frequently begin with trainers asking questions to test CHWs’ knowledge, which can be difficult for CHWs who fear being wrong. Sharing memories, on the other hand, allows a CHW to talk about her own experiences, which are neither right nor wrong and do not require recall of something previously learned. In addition, the safe workshop environment provides each CHW with the opportunity to express herself. In this process, trust and empathic bonding develop among CHWs-in-training and trainers, especially when memories are emotionally charged. This circumstance increases the likelihood that they will collectively adjust to a new way of thinking and doing things, and it promotes mutual social support to sustain new behaviors. Furthermore, a person’s self-esteem is strengthened when they understand and take ownership of their cultural beliefs. Research on autobiographical memory suggests that a person’s negative or “low closure” experiences have more effect on future behavior than positive or “high closure” experiences because negative memories maintain a stronger emotional charge and their details are more present in memory.^39^ Second-hand memories, heard from others in a group, can affect future behavior similar to one’s own memories. Through first sharing histories in a group and then learning new ways to do things from a trainer, CHWs seemed to gain enthusiasm about sharing new knowledge in a similar way to convince mothers.

Women’s personal experiences with childbearing and child rearing are generally not valued, but SH specifically recognizes and builds on them.

A potential effect modifier of our results on stunting was the discontinuation of the National Feeding Program government food supplementation program for children ages 6 months to 3 years, that occurred half-way through the 4-year study. We postulate that it could have contributed to the plateauing of stunting prevalence from baseline to endline in the control group (Figure 3). The reduction of stunting in both study groups could arguably have been greater without this effect.

We speculate that some of the significant improvement in knowledge of danger signs and reported child feeding practices in both study groups was due to the use of the same training materials (flip charts) for all CHWs. Particularly considering the illiterate mothers in our study, their significant knowledge and reported behavior improvements did not carry through to improved growth of their children. High prevalence of stunting at 40% or above at both baseline and endline among children of illiterate mothers in both study groups may be partially explained by so-called environmental enteric dysfunction.^60^ We surmise that stunting in this population could not be overcome by the adoption of the specific behaviors that were promoted and measured. This explanation is supported by 2 recent major trials. A cRCT in rural Zimbabwe tested interventions to reduce stunting by addressing environmental contamination either alone or in combination with infant and young child nutritional improvements. No reduction in stunting was found except when a food supplement was provided along with nutritional counselling.^61^ Another cRCT in Bangladesh using direct counseling combined with a mass media campaign found improved feeding practices with no improvement in growth.^62^

Finally, our finding that our experimentally trained CHWs tended to visit less-educated mothers more, especially those with only primary education (Tables 4 and 7), is consistent with a recent systematic review of factors contributing to equity of CHW services that suggests CHWs tend to visit more vulnerable mothers.^63^

### Limitations

The main limitation of our study design was the small number of study clusters, which was constrained by the number of available clusters in the study area, defined as PHC facility jurisdictions. Matching and randomization of study clusters, triple-blinding, use of DID and GEE for data analysis, and adjustment for covariates were design or analysis factors that strengthened power and reliability of the study findings.^64^ Our study did not measure all potential predictive factors for stunting.

## CONCLUSIONS

Our study suggests that the SH method for training community health workers (CHWs) was associated with reduced child stunting when mothers were literate. Regardless of educational level, many women who live in traditional societies with culturally rooted beliefs may have special needs for effective learning for maternal and child health and nutrition. These women could be reached with behavior change strategies through CHWs trained with methods such as SH. Efforts to prevent stunting with such a behavior change strategy could be prioritized in the “low-hanging fruit” of children of women with at least some education to quickly reduce global stunting rates. The poorest children have many other determinants of stunting that are more challenging but important to address.

This study extends the research on CHW program implementation. To our best knowledge, this randomized trial is the first to test the application of autobiographical memories to help women be empowered with capabilities to serve as change agents with other women in their communities. Our study may also represent an application of neurological findings on the physical consolidation of neurons by building on memory recall to strengthen educational interventions.

More effective community health promotion is needed to attain better health outcomes in LMICs. We suggest that an integrated system should focus on strengthened government services that support and sustain careful CHW selection by women in communities, methodologically sound CHW training based on autobiographical memories used in conjunction with visual teaching material, consistent community-based supportive supervision of female CHWs, and effective monitoring and evaluation. Further implementation research on best ways to strengthen these conditions for the deployment of CHWs can contribute to meeting the basic health rights of mothers and children in LMICs and to reaching the Sustainable Development Goals.

**Figure:**
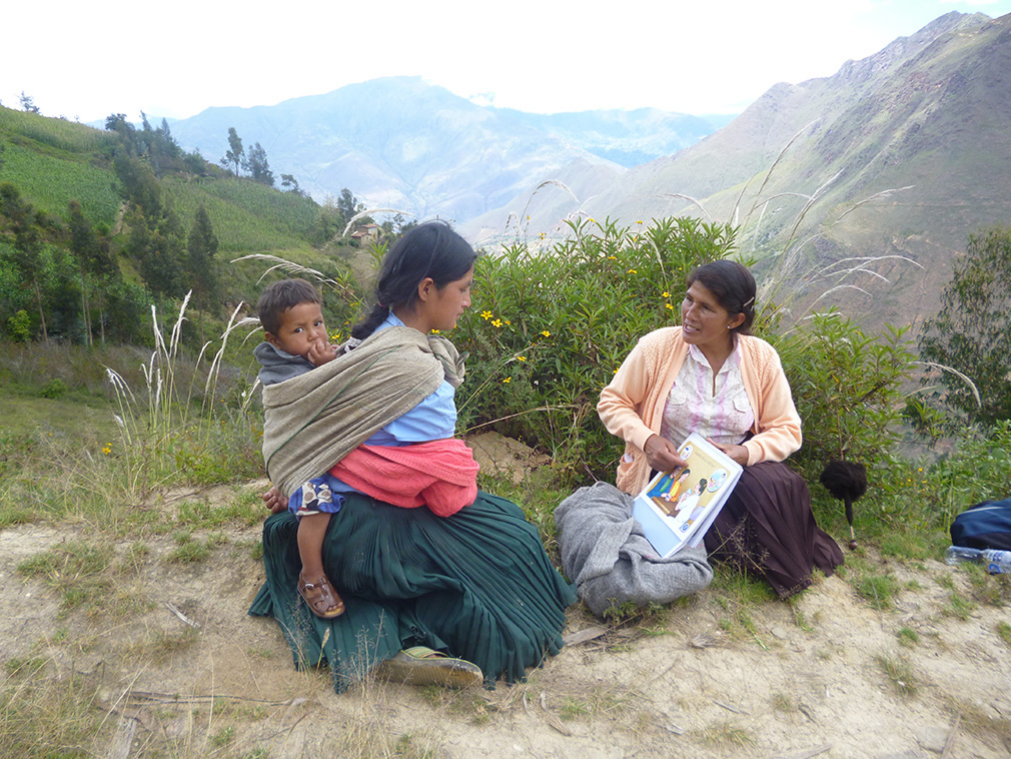
Community health worker sharing histories with mother and reviewing flip chart messages.Credit: ©2014 Lurdes Cabello/Future Generations

**Figure:**
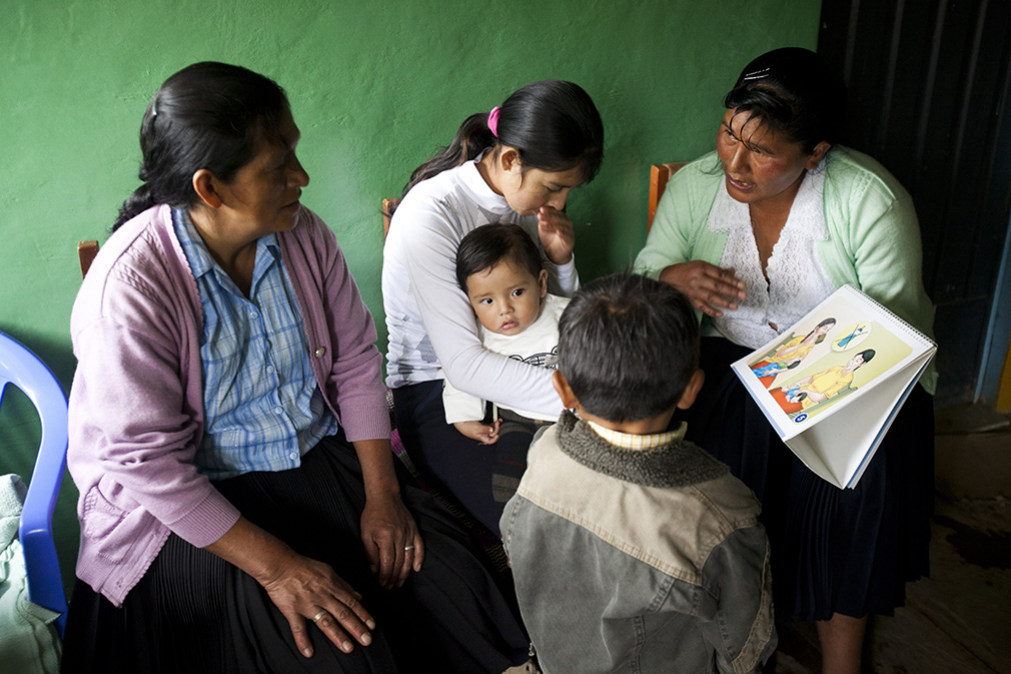
Community health worker asks a mother and grandmother what they see in the flip chart image. Credit: ©2013 Elie Gardner

**Figure:**
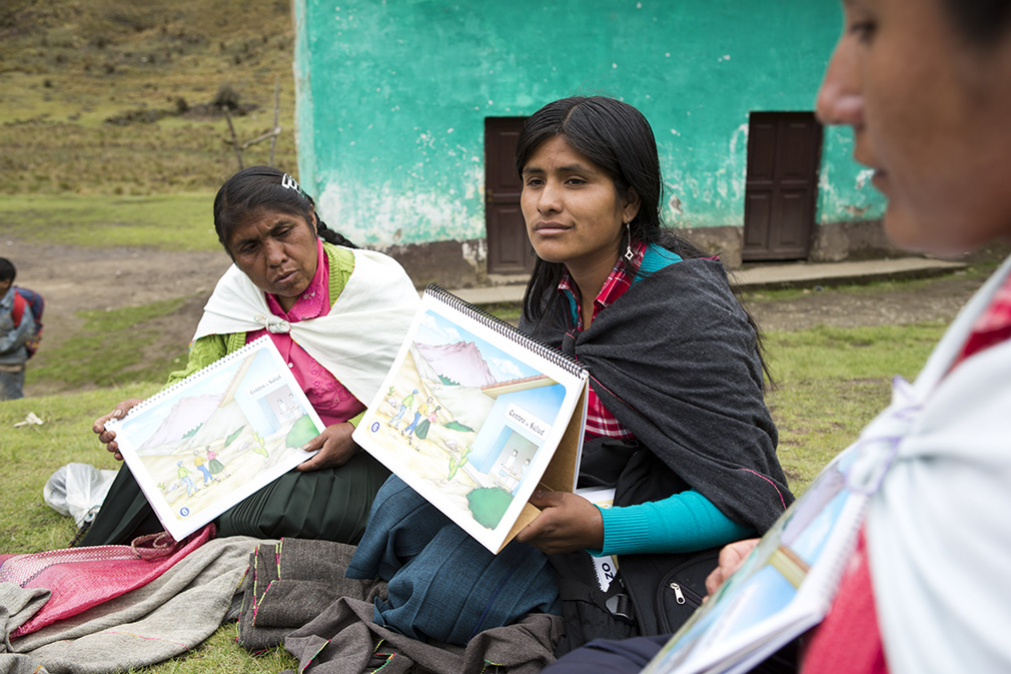
Community supervisor meeting with small group of community health workers to reinforce training received from health personnel trainer that month. Credit: ©2013 Elie Gardner

## Supplementary Material

19-00332-Altobelli-Supplement.pdf

## Notes

**En español**

**Ensayo aleatorizado por clústeres para probar Compartiendo Historias como un método de capacitación para agentes comunitarias de salud en el Perú**

**RESUMEN**

**Antecedentes:** Las agentes comunitarias de salud (ACS) se están desplegando cada vez más para apoyar la adopción por parte de las madres de prácticas saludables en el hogar. Sin embargo, se sabe poco en cuanto a la mejor manera de capacitarlas para que tengan las capacidades y competencias culturales necesarias para apoyar el cambio de comportamiento en salud de las madres. Probamos nuestro nuevo método de entrenamiento de ACS, Compartiendo Historias, en el que las ACS relatan sus experiencias de gestación, nacimiento y crianza de sus propios hijos, sobre las que se construye un nuevo aprendizaje.

**Métodos:** Realizamos un ensayo aleatorizado en clústeres en el Perú rural en 18 clústeres apareados. Cada clúster era el ámbito de un establecimiento de salud del primer nivel de atención. El personal de salud capacitó a las ACS con Compartiendo Historias (clústeres experimentales) o un método de capacitación estándar (clústeres de control). Todas las demás intervenciones de capacitación y fortalecimiento del sistema fueron iguales entre los dos brazos del estudio. Todas las ACS llevaron a cabo visitas domiciliarias con mujeres embarazadas y niños de 0 a 23 meses para enseñar, monitorear las prácticas de salud y los signos de peligro, y referirlas. El resultado principal fue baja talla-por-edad menos de -2 Z scores en niños de 0 a 23 meses. Encuestas de hogares repetidas se llevaron a cabo al inicio del estudio como línea de base (606 casos) y en la evaluación final después de 4 años (606 casos).

**Resultados:** Las características de las madres y sus hijos fueron similares tanto en la línea de base como en la evaluación final. El análisis de diferencias-en-diferencias (DiD) mostró que los cambios promedios de talla-por-edad no fueron significativamente diferentes en los clústeres experimentales frente a los de control desde la línea de base hasta la línea final (*P=*.469). Sin embargo, en el subgrupo de madres que pueden leer (alfabetas), la talla-por-edad mejoró en 1.03 en la escala de Z-score en los clústeres experimentales en comparación con los clústeres de control desde la línea de base hasta la línea final (*P=*.059). Asimismo, usando ecuaciones de estimación generalizadas (GEE), demostramos que la desnutrición crónica en los hijos de las madres alfabetas se redujó significativamente (Beta=0,77; intervalo de confianza del 95% 0,23, 1,31; *P*<.01), controlando adjustando por covariables.

**Conclusión:** En comparación con los métodos de entrenamiento estándar para ACS, Compartiendo Historias puede haber mejorado la eficacia de las ACS como agentes de cambio entre las madres alfabetas para reducir la desnutrición crónica infantil. La baja talla-por-edad experimentada por los hijos de madres analfabetas puede implicar determinantes del retraso en el crecimiento que no fueron abordados en este estudio.

**Peer Reviewed**

**First published online:** December 11, 2020

**Funding:** This field project with embedded cluster randomized controlled trial research was made possible by a competitive grant to Future Generations from the United States Agency for International Development – Office of Global Health (Child Survival and Health Grants Program, Cooperative Agreement No. AID-OAA-10-00048) for the project Health in the Hands of Women: A Test of Teaching Methods in addition to support from the Carl E. Taylor Endowment for Equity and Empowerment, Future Generations University, and local government partners in Peru. The Maternal Health Task Force of EngenderHealth through a subgrant from the Bill and Melinda Gates Foundation (GMH-107-1) provided support to an earlier project in indigenous Andean villages of Cusco to adapt the sharing histories method to Peru. The views expressed in this article are the authors’ and are not an official position of the funder.
