# Supplementary material for: A Cluster-Randomized Trial to Test Sharing Histories as a Training Method for Community Health Workers in Peru
Source: Glob Health Sci Pract. 2020 Dec 23;8(4):732–58. doi: 10.9745/GHSP-D-19-00332 (PMC7784074; doi:10.9745/GHSP-D-19-00332)
Supplement: 19-00332-Altobelli-Supplement.pdf [file 19-00332-Altobelli-Supplement.pdf]

Supplement to: Altobelli LC, Cabrejos-Pita J, Penny M, Becker S. A cluster randomized trial to test Sharing Histories as a training method for community health workers in Peru. *Glob Health Sci Pract*. 2020;8(4). https://doi.org/10.9745/GHSP-D-19-00332

Supplement

"Health in Hands of Women Project" in Huánuco, Peru With Embedded cRCT

I. MONITORING DATA ON CHW TRAINING: Workshop Attendance and Pre- and Post-Test Scores

a. CHW workshop attendance rates for six sets of workshops by module topic

Attendance rates at monthly workshops were 82% or higher for five of the six module topics, as shown in Table A. These were the workshops provided for community health workers (CHW) by a trained health care provider in the primary health care facility. However, these rates do not fully reflect the learning by CHW. For CHW who missed a workshop, community supervisors met with the CHW in their community to reteach the module content.

Table A: Attendance of CHW at monthly training workshops at the health facility by module topic

**Training Module Topic**	**Control**		**Experimental**		**P value**
	N°	%	N°	%	
Pregnancy	271	100%	235	100%	ns
Birth & Postpartum	222	82%	200	85%	ns
Newborn	239	88%	229	97%	p<.05
Breastfeeding	236	87%	198	84%	ns
Diarrhea	226	83%	193	82%	ns
Infant Growth & Nutrition	199	73%	149	63%	p<.05

Source: Prepared by Vilma Suárez, Health in Hands of Women: A Test of Teaching Methods Project, Future Generations.

b. Pretest and posttest scores for each module topic

We applied knowledge tests to each CHW immediately before and after each training module. Tests were applied verbally to CHWs in a private setting with responses noted by the evaluator who was usually the health care provider trainer. CHW scores showed highly significant improvements from pretest to posttest for all module topics.

The result of community follow-up training can be seen in the posttest scores for diarrhea and infant growth & nutrition which are higher than those of other module topics (Table B), even though their workshop attendance was less than for the other topics. This was especially true for the experimental group as compared to the control group.

Supplement to: Altobelli LC, Cabrejos-Pita J, Penny M, Becker S. A cluster randomized trial to test Sharing Histories as a training method for community health workers in Peru. *Glob Health Sci Pract*. 2020;8(4). https://doi.org/10.9745/GHSP-D-19-00332

Table B: Pretest and Posttest Scores of CHW for each training module topic

**Training Module Topic**	**Pretest Scores***				**Posttest Scores**				**Difference in scores Pretest to Posttest**	
	**Control**		**Experimental**		**Control Group**		**Experimental**		**Con.**	**Exp.**
	# CHW	Score	#CHW	Score	#CHW	Score	#CHW	Score	P value	P value
Pregnancy	162	46.1	183	51.5	216	83.2	209	80.8	<.001	<.001
Birth&Postpartu	171	41.9	105	39.2	127	81.1	197	76.6	<.001	<.001
Newborn	125	48.2	130	46.1	216	83.3	183	79.0	<.001	<.001
Breastfeeding	92	44.3	123	41.3	158	83.0	177	82.1	<.001	<.001
Diarrhea	160	69.2	124	69.5	180	73.9	145	96.3	<.001	<.001
Growth & Nutrition	98	25.3	135	32.2	141	82.2	142	90.0	<.001	<.001
Adjusted Mean		47.38		46.97		81.18		83.29	<.001	<.001

*Score = % of test items answered correctly.
Source: Prepared by Vilma Suarez and Laura C. Altobelli, Health in Hands of Women: A Test of Teaching Methods Project, Future Generations.

Supplement to: Altobelli LC, Cabrejos-Pita J, Penny M, Becker S. A cluster randomized trial to test Sharing Histories as a training method for community health workers in Peru. *Glob Health Sci Pract*. 2020;8(4). https://doi.org/10.9745/GHSP-D-19-00332

II. COST INFORMATION ON THE COMMUNITY HEALTH INTERVENTION FOR SCALING-UP BY THE GOVERNMENT

The principal investigator did a detailed cost estimate for the effective introduction of this full integrated community health model into a government primary health care (PHC) system. Costs are relatively low based on the assumption that Ministry of Health (MOH) staff will be involved as program supervisors and CHW trainers at no additional salary cost. The model assumes involvement of local municipalities in financial support of stipends for community supervisors (CS) as well as non-financial incentives for CSs and CHWs.

Therefore, cash costs to be assumed by the MOH to implement the model include:

(1) training costs for trainers, CSs, and CHWs;
(2) start-up cost of reproducing training manuals and flipcharts for eight modular topics given as job aids to trainers, CHWs, CSs and each PHC facility for counseling mothers;
(3) on-going costs of reproducing monitoring and supervision forms; and
(4) the partial cost of external human resource involvement which is limited to one expert training consultant to train MOH trainers for a small proportional of her time- effort in one district based on population size of the district.

<u>Results</u>

For a district of 25,000 inhabitants and seven PHC facilities, cost per child under age five would be USD <u>$16.57</u> per child for the first year to start up. The cost is divided into $11.37 from the MOH and $5.20 for the local government contribution for community supervisor stipends. Annual maintenance costs per child under age 5 would be reduced to USD <u>$14.55</u> divided between $9.35 from the MOH and $5.20 for the local government contribution. This cost includes one full-time MOH staff person per district in charge of managing the community health program, paid by the MOH; in addition, a stipend will be paid to 10 CSs by the municipal government.

Local in-kind resources not included in the costs are the percent effort of a health service network (*Red de Salud* in Spanish) supervisor, 10 health staff who serve as CHW/CS trainers, 167 CHWs, and 45 PHC staff who receive orientation-training to support the community health model.

For a district of 10,000 inhabitants with 3 PHC facilities and with fewer children <5 years, the estimate is marginally more at $17.34 per child per year total. The alternative cost is evidently much lower and close to zero because there is no community health program to cost out.

One could consider $16.57 (start-up) and $14.55 (maintenance) per child per year, or slightly over $1.00 per month, as expensive in one country but not in another. Costs would also vary by country according to the local cost of inputs.

Current practice is to have no community health program, or one that is in place but is not effective. In our case, the cost was the same for the model in both study groups. The experimental training clusters had better results than the other due to better training methods, but these did not increase the cost.

Supplement to: Altobelli LC, Cabrejos-Pita J, Penny M, Becker S. A cluster randomized trial to test Sharing Histories as a training method for community health workers in Peru. *Glob Health Sci Pract*. 2020;8(4). https://doi.org/10.9745/GHSP-D-19-00332

SUMMARY - COST OF COMMUNITY HEALTH PROMOTION PROGRAM WITH CHW BY DISTRICT SIZE

COSTED ITEMS	District of 25,000 inhabitant and 7 PHC facilities	District of 10,000 inhabitants and 3 PHC facilities
	N° of units	N° of units
**Training costs calculated on basis of # persons:**		
Health personnel - Sectoristas	45	18
Health personnel - Trainers	10	4
Community supervisors	10	4
CHW - Women Leaders	167	67
**Salary - Technical Assistant for district**	1	0.5
**Stipend - Community Supervisors**	10	4
**Educational material**		
Flipcharts		
Facilitator manuals for trainers		
**Health network supervisor (% of time)**	100%	30%
**Training Specialist**	30%	10%
Number of children 0-4 years	3,000	1,200
**TOTAL COST TO ESTABLISH THE PROGRAM YEAR 1**		
Health Salud	S/. 110,859	S/. 48,353
Local government	S/. 50,655	S/. 19,260
**TOTAL**	S/. 161,514	S/. 67,613
**Total annual cost per child 0-4 years (soles)**	**S/. 54**	**S/. 56**
Annual cost Health Sector	S/. 37	S/. 40
Annual cost local government	S/. 17	S/. 16
**Total annnual cost per child 0-4 yrs. (USD)**		
**Exchange rate: S/3.25 soles per 1 USD**	**$16.57**	**$17.34**
Cost for health sector per child < age 5	$11.37	$12.40
Cost for local government per child < age 5	$5.20	$4.94
**ANNUAL MAINTANENCE COST AFTER YEAR 1**		
For health sector	91,159	
For local government	50655	
TOTAL	141,814	
**Total maintenance cost per child < age 5 year**	**S/. 47**	
Cost for health sector per child < age 5	S/. 30	
Cost for local government per child < age 5	S/. 17	
**Total cost per child 0-4 yrs. (USD)**	**$14.55**	
Cost for health sector per <5	$9.35	
Cost for local government per <5	$5.20	

Prepared by Laura C. Altobelli, Nov. 2020

Supplement to: Altobelli LC, Cabrejos-Pita J, Penny M, Becker S. A cluster randomized trial to test Sharing Histories as a training method for community health workers in Peru. *Glob Health Sci Pract*. 2020;8(4). https://doi.org/10.9745/GHSP-D-19-00332

COSTS OF COMMUNITY HEALTH PROMOTION MODEL FOR A DISTRICT

					District 25,000 inhabitants & 7 PHC facilities			District 10,000 inhabitants & 3 PHC facilities		
**Actor / Concepto**	**Annual Unit cost training**	**Annual Unit cost materials**	**Annual Unit cost operations (transport, per diem)**	**TOTAL Annual cost per person**	**# units**	**TOTAL MOH**	**TOTAL Local Government**	**# units**	**TOTAL MOH**	**TOTAL Local Government**
**PHC health staff**										
Training on Sectorization	0	0	0	0						
TOTAL				**0**	45	S/. 0		18	S/. 0	
**Health Personnel Trainers of CHW**										
Training on Sharing Histories and Adult education methods	60	0	100	160						
Training as trainer - 8 modules - 1 day per module	80	0	200	280						
Monthly workshop for CHW/CS	48	0	0	48						
Set of training materials - 8 facilitator manuals & 7 flipcharts r y 7 rotafolios	0	200	0	200						
TOTAL				**688**	10	S/. 6,880		4	S/. 2,752	
**Community Supervisor**										
Monthly training in PHC facility	60	0	24	84						
Set of training material - 7 flipcharts	0	100	0	100						
TOTAL MOH				**184**	10	S/. 1,840		4	S/. 736	
Incentive from local government	0	15	0	15						
Stipend from local government	0	0	4800	4800						
TOTAL LOCAL GOVERNMENT				**4815**	10		S/. 48,150	4		S/. 19,260
**Community Health Worker (CHW) Women Leader**										
Monthly training in PHC facility	60	0	24	84						
Set of training material - 7 flipcharts	0	100	0	100						
TOTAL MOH				**184**	167	S/. 30,728		67	S/. 12,328	
Incentive from local government	0	15	0	15						
TOTAL LOCAL GOVERNMENT				**15**	167	S/. 2,505	S/. 2,505			
**FIXED PERSONNEL COST**										
**Technical assistant - 1 per district of 25,000 inhabitant and 6 PHC facilities**										
Full time as trainer and supporter	54000	0	0	54000						
Transport and per diem	0	0	3850	3850						
**TOTAL**				57850	1	S/. 57,850		0.5	S/. 28,925	
Community Health Supervisor - Network	0	0	2200	2200	1	S/. 2,200		0.3	S/. 660	
**FIRST YEAR EXPENSE**										
Training specialist	14520	9000	6000	29520	0.3	S/. 8,856		0.1	S/. 2,952	
**COSTO ANUAL POR DISTRITO**						**S/. 110,859**	**S/. 50,655**		**S/. 48,353**	**S/. 19,260**

Prepared by Laura C.Altobelli Nov. 2020

Supplement to: Altobelli LC, Cabrejos-Pita J, Penny M, Becker S. A cluster randomized trial to test Sharing Histories as a training method for community health workers in Peru. *Glob Health Sci Pract*. 2020;8(4). https://doi.org/10.9745/GHSP-D-19-00332
